# Modeling and Experimental Validation for Detecting Indoor Respiratory Droplet

**DOI:** 10.3390/bios16090492

**Published:** 2026-09-03

**Authors:** Fuqiang Hu, Pengfei Zhang, Yusheng Sun, Xiaofang Wang, Pengfei Lu

**Affiliations:** College of Information Science and Technology, Shihezi University, Shihezi 832003, China; 20242108056@stu.shzu.edu.cn (F.H.); 20232108002@stu.shzu.edu.cn (P.Z.); 20232108025@stu.shzu.edu.cn (Y.S.)

**Keywords:** droplet transmission dynamics, multi-scale modeling, experimental validation, ventilation strategies, molecular communication testbed, biosensing, diagnostics

## Abstract

Precise quantification of respiratory pathogen transmission is urgently needed to underpin disease surveillance and support diagnostic decision-making in indoor healthcare settings. Although numerical simulations are widely used to study macro-scale transmission, the key physical parameters governing droplet dynamics remain insufficiently understood. This study develops a multi-scale transmission model to evaluate the effects of droplet evaporation, sedimentation, and ventilation on viral transmission. Based on the Wells evaporation–sedimentation theory, a time-varying model is formulated incorporating droplet size distribution, environmental humidity, and ventilation conditions, with analytical expressions derived for concentration distributions across different respiratory activities (breathing, speaking, coughing, and sneezing). Results show that droplet size is decisive for transmission distance and lower relative humidity significantly extends sedimentation range. Small coughing droplets can travel up to 2.5 m, while the bimodal sneezing distribution (1.5 µm and 74 µm) generates high-concentration zones up to 2 m. To validate the model, a molecular communication testbed is developed as a biosensing-oriented platform integrating transmitters, receivers, and configurable ventilation modules with real-time sensing capabilities, enabling systematic verification under varied breathing modes, humidity, and ventilation conditions. Experimental results show strong agreement with theoretical predictions. This framework provides a quantitative basis for biosensing-enabled environmental monitoring and diagnostic-oriented risk assessment, informing ventilation optimization and infection control measures in indoor environments.

## 1. Introduction

Respiratory disease transmission poses a serious threat to human health [1,2]. Understanding the mechanisms underlying viral spread is therefore essential for developing effective prevention strategies and reducing the global burden of respiratory diseases. As an effective means of acquiring biological information, biosensing has become a core technology in medical diagnostics, and biosensing-based detection of airborne pathogens provides critical guidance for early and rapid diagnosis as well as indoor disease surveillance. Airborne transmission is a primary route for many respiratory infections, especially in confined or poorly ventilated environments, complicating the precise measurement and control of epidemic spread [3,4,5,6,7]. Even in spaces without mechanical ventilation, natural air movements driven by temperature gradients, occupant activity, and door openings can sustain airborne pathogen transport over significant distances [8,9]. Ventilation therefore plays a decisive role in modulating transmission risk: case studies in poorly ventilated settings (Guangzhou restaurant at 1 L/s per person [9,10], Skagit Valley choir at 0.3–1 ACH [11,12]) demonstrate that inadequate air exchange facilitates aerosol accumulation and superspreading events, while extensive computational and experimental investigations have established that properly designed ventilation systems substantially reduce exposure [13,14]. Recent laser-based visualization studies conducted by the NIH and the University of Pennsylvania showed that speaking generates numerous droplets with radii ranging from 20 to 500 μm [15,16]. Different respiratory activities (speech, coughing, sneezing) produce distinct airflow speeds and turbulence, which determine the atomization, droplet counts, and size distributions [17,18,19,20]. After exhalation, droplets partially or fully evaporate, or reach an evaporation–condensation balance, forming aerosol nuclei [21]. These nuclei can serve as efficient carriers of viruses [22,23]. Their sizes depend on droplet composition, initial radius, and environmental conditions [24,25], which together govern indoor transport and deposition [8,26,27,28,29]. Larger droplets settle quickly and drive short-range “droplet transmission,” while smaller droplets tend to evaporate into aerosol nuclei that travel farther with airflow, enabling “aerosol transmission.”

Case studies underscore the critical role of ventilation in airborne transmission. In a Guangzhou restaurant outbreak, one index case infected 10 others without direct contact; airflow simulations supported aerosol transmission under a per-person ventilation rate of only 1 L/s [9,10]. During the Skagit Valley Choir event, 52 of the 61 attendees were infected despite precautionary measures; the low measured ventilation rate of 0.3–1 ACH likely facilitated aerosol spread and increased exposure [11,12]. These observations emphasize ventilation as a critical engineering control for mitigating indoor transmission [13].

Recent computational studies on aerosol transport and deposition have further advanced the understanding of droplet dynamics in indoor environments. For instance, Wang et al. [30] investigated the deposition characteristics of ultrafine particles of different shapes in inertial impactors using CFD–DEM simulations, while Xu et al. [31] numerically examined the effects of exposure conditions and human movement on cough droplet transmission and deposition. These studies highlight the importance of incorporating particle-scale physics and realistic boundary conditions into transmission models, thereby motivating the development of a multiscale framework that integrates droplet evaporation, sedimentation, and ventilation effects within an analytically tractable propagation model.

Substantial progress has been made in understanding indoor aerosol transmission through combined computational and experimental approaches [8,13,14]. Many studies rely on macroscopic statistics limited by measurement sensitivity, providing only limited insight into the coupled microscale processes of evaporation, sedimentation, and airflow. Fluid-dynamics-based modeling provides a path to mechanistic understanding [32,33,34,35]. Notably, coughing behaves as a transient, high-concentration source, whereas breathing acts as a continuous, low-concentration source [36]. Yet, most existing models do not jointly integrate droplet physics (size evolution, evaporation) with environmental factors (humidity, ventilation), limiting robust assessment of transmission risk and control strategies. Moreover, although computational models provide valuable theoretical insights, their applicability to engineering practice remains limited by the lack of systematic experimental validation on controlled physical testbeds. The integration of theoretical modeling with cyber–physical experimental platforms represents a promising approach to bridge this gap, enabling both model validation and the development of data-driven control strategies for indoor air quality management.

**Figure 1:**
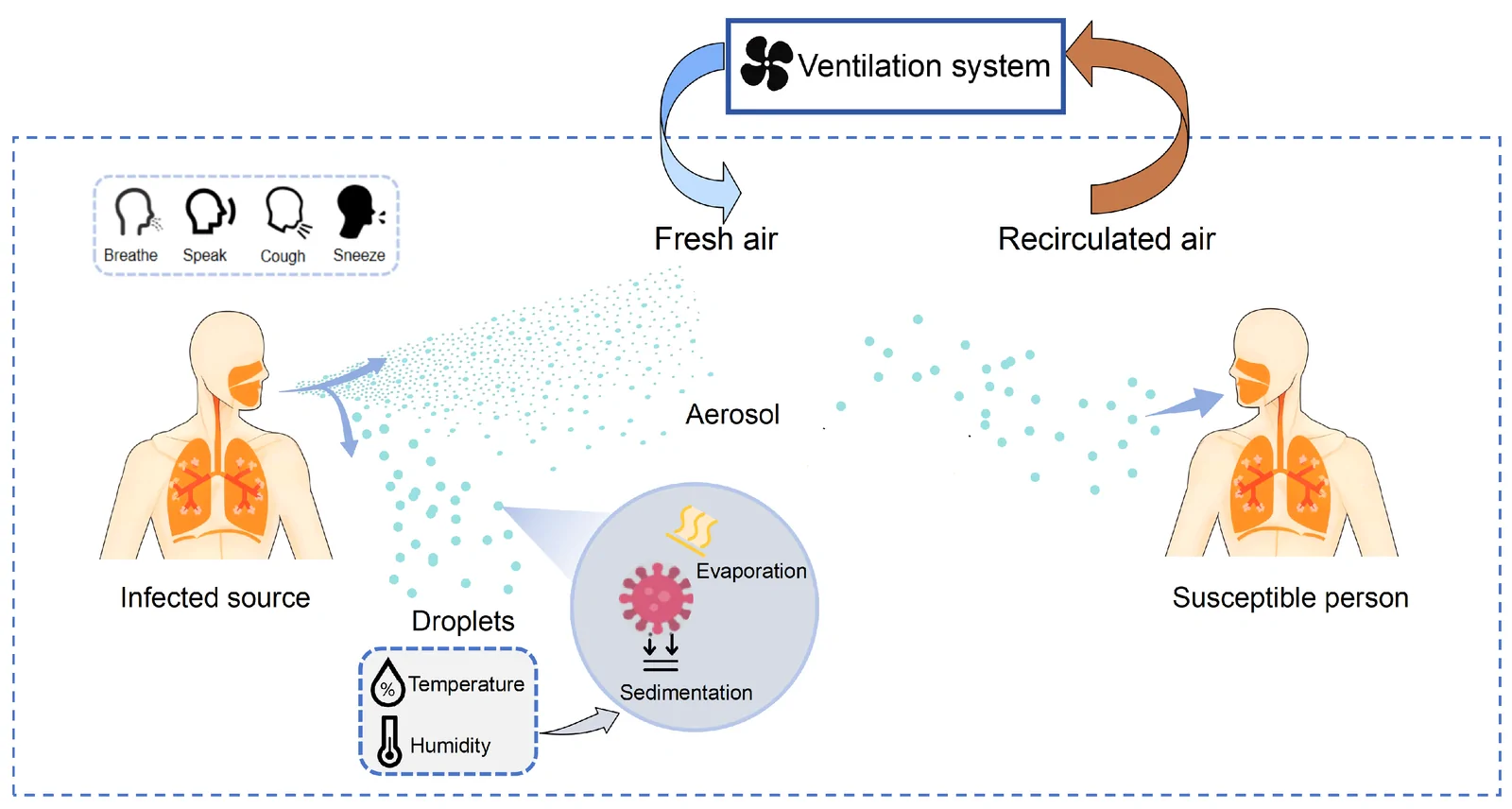
Schematic diagram illustrating the transmission route of respiratory viral droplets and the intervention of the ventilation system. This transmission process includes expulsion from the infected source, airborne spread, and inhalation by susceptible individuals.

To bridge this gap, we propose a multi-scale viral transmission model that integrates droplet physical factors with dynamical theory, as depicted in Figure 1. Different from existing analytical models that treat individual physical processes (evaporation, drag, or diffusion) in isolation, the proposed framework couples these processes into a unified time-dependent propagation model that explicitly accounts for temporal evolution of droplet radius, velocity, and spatial concentration under different respiratory activities and ventilation strategies. This integrated approach enables the analytical derivation of closed-form concentration expressions across multiple respiratory modes—breathing, speaking, coughing, and sneezing—within a single consistent formulation. The model quantifies key processes including droplet forces (gravity and drag), evaporation, and deposition, and analyzes the impact of different respiratory modes (speaking, coughing, sneezing) and ventilation strategies (mixed ventilation (MV), displacement ventilation (DV), and downward ventilation (DWV)) on transmission risk. We further discuss strategies for computing concentrations at susceptible locations within the scenario. In addition, we develop and deploy a horizontal molecular communication testbed to experimentally validate the theoretical framework, providing a cyber–physical platform for assessing droplet transmission dynamics under controlled conditions. The model considers a simplified indoor geometry without furniture, occupants, or thermal sources beyond the infected individual; these idealizations, while necessary for analytical tractability, may overestimate or underestimate local concentrations in realistic settings, and their implications are discussed further in Section 5. Additionally, breathing and speaking are treated as continuous steady-state sources, whereas coughing and sneezing are modeled as instantaneous pulsed releases—consistent with established classifications in the literature [36,37]. The model does not resolve turbulent eddy structures directly; instead, turbulent dispersion is captured through an effective diffusion coefficient $D_{\mathrm{tu}}$, an approach widely adopted in analytical indoor aerosol models [28]. The specific contributions are as follows:

1. We develop a dynamical modeling framework for virus-laden droplet transmission that incorporates mechanisms such as droplet evaporation, deposition, and ventilation effects into a time-evolving propagation model. We explicitly consider and quantify the influence of droplet radius, velocity, and evaporation characteristics on transmission.
2. We analyze how spatial concentration and the exposure concentration of susceptible individuals vary under different respiratory activities and ventilation strategies, and we assess the magnitude of their impacts on concentrations.
3. We design and implement a molecular communication testbed that serves as a biosensing-enabled physical validation platform, enabling systematic experiments to verify the distance–concentration relationship under different breathing modes, humidity conditions, and ventilation strategies. The testbed integrates signal modulation, environmental control, and real-time biosensing capabilities, bridging the gap between theoretical modeling and practical engineering applications for indoor pathogen monitoring.
4. We validate the effects of the considered factors on spatial concentration and exposure concentration of susceptible individuals through both numerical simulations and physical experiments, and we offer mitigation recommendations based on ventilation optimization and behavioral interventions applicable to the design and management of indoor built environments.

The remainder of this paper is organized as follows: Section 2 details the dynamical modeling of droplet transmission; Section 3 presents and discusses the numerical simulation results; Section 4 describes the experimental testbed design and validates the theoretical model through systematic experiments; Section 5 provides further discussion.

## 2. Theoretical Model

Airborne transmission of respiratory viruses primarily occurs when infected individuals engage in activities such as coughing, sneezing, speaking, and breathing, which release virus-laden droplets into the surrounding air. These droplets may be inhaled or come into contact with nearby individuals, leading to potential infections [38]. The number, velocity, and size distribution of droplets vary depending on the nature of the respiratory event. During transport, droplets experience aerodynamic drag whose magnitude depends on the Reynolds number. Additionally, droplets are affected by gravity, evaporation, and other factors, with their impacts being dependent on specific droplet properties and environmental conditions such as droplet radius, relative humidity (RH), solute concentration, and density. By incorporating these elements into the equations of motion for droplets, the resulting effects can be quantitatively represented in concentration equations.

### 2.1. The Effect of Droplet Evaporation on Radius

**Figure 2:**
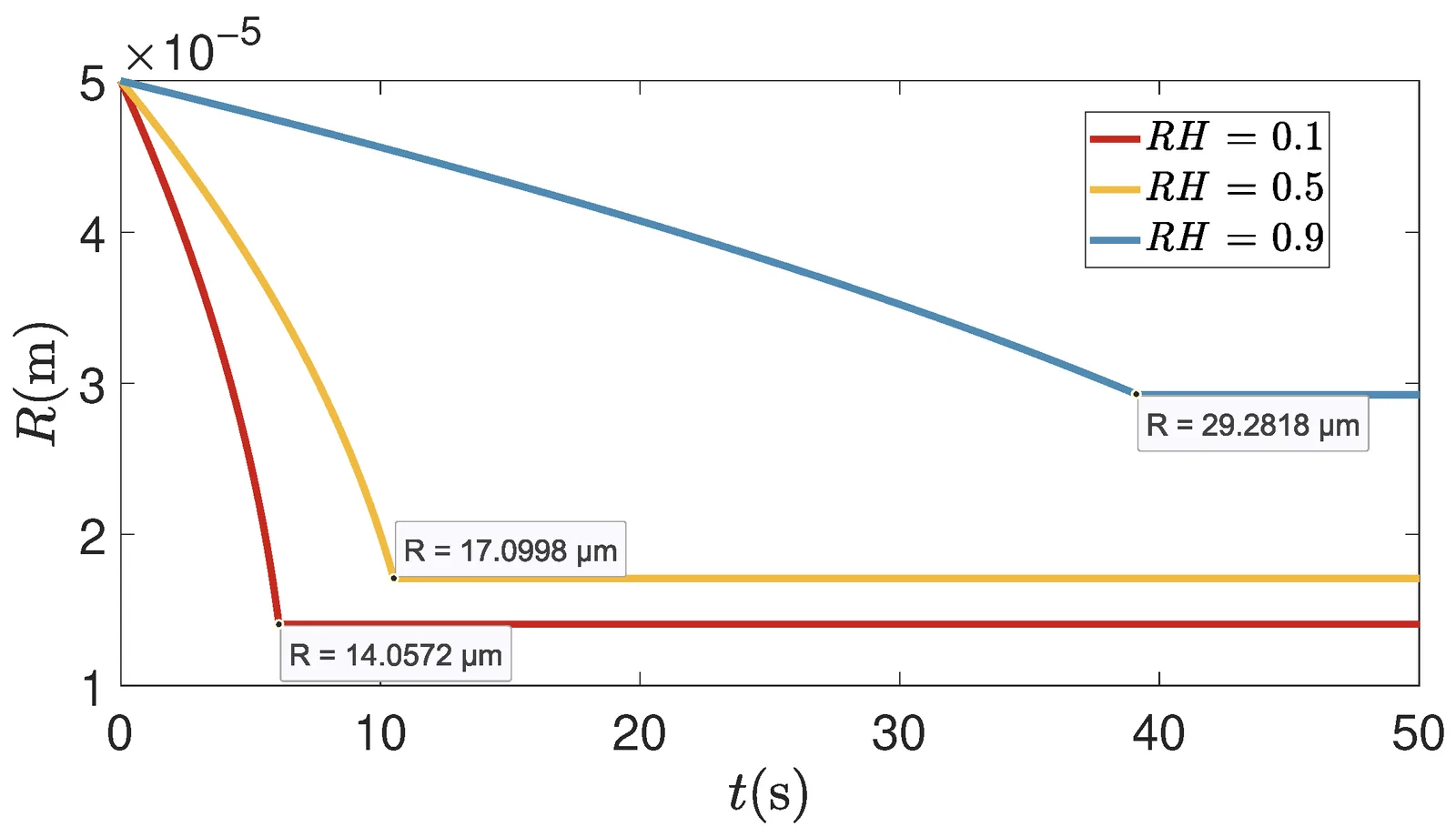
Evolution of droplet radius *R* over time *t* for 50 µm droplets at three different RH levels.

Evaporation plays a critical role in droplet transmission and is largely influenced by the RH of the surrounding air and the initial droplet size. The classical Wells evaporation-settling model [21] first clarified the relationship between evaporation and droplet size. This prediction has been further confirmed by multiple studies [24,39,40]. As a droplet evaporates, water is continuously lost from its surface, causing its radius to decrease gradually. The relationship between the droplet radius R(t) and time *t* during evaporation can be expressed as

(1) $$\begin{array}{c} t=\frac{R_{0}^{2}}{\theta (1-\mathrm{RH})}\left[1-\frac{R{\left(t\right)}^{2}}{R_{0}^{2}}\right], \end{array}$$

where $R_{0}$ represents the initial droplet radius, RH denotes the relative humidity of the environment, and θ is a constant ($\theta =4.2\times 10^{-10}\;m^{2}/s$). Solving for R(t) yields $R\left(t\right)=\sqrt{R_{0}^{2}-t\theta \left(1-\mathrm{RH}\right)}$. By substituting R(t) into the corresponding subsequent equations, the variation in droplet radius during evaporation can be characterized, as illustrated in Figure 2.

As shown in the figure, the droplet radius decreases due to evaporation at a rate governed by the difference between the saturated vapor pressure at the droplet surface and the ambient water vapor partial pressure. At high RH levels (e.g., RH = 0.9), the ambient vapor pressure approaches the vapor pressure at the droplet interface, reducing the vapor pressure gradient and, consequently, the evaporation driving force. From a mass-transfer perspective, a higher RH reduces the water vapor concentration gradient around the droplet, thereby decreasing its evaporation rate. Additionally, as RH increases, the droplet equilibrium temperature approaches the ambient temperature, leading to further reductions in evaporation rates [22,41].

As evaporation progresses, the droplet radius gradually converges to an equilibrium value (for RH = 0.1, R=14.0572 µm; for RH = 0.5, R=17.0998 µm; and for RH = 0.9, R=29.2818 µm). The time required to reach this equilibrium increases with higher RH. This equilibrium value is reached when the volatile liquid has evaporated, leaving behind a solid, nonvolatile residue. In 1934, Wells first referred to this value as the “droplet nucleus” radius [21]. The equilibrium radius, denoted as $R_{\mathrm{ev}}$, is given by [42,43]

(2) $$\begin{array}{c} R_{\mathrm{ev}}=R_{0}{\left(\frac{\Phi_{0}}{1-\frac{\mathrm{RH}}{\gamma }}\right)}^{\frac{1}{3}}, \end{array}$$

where γ denotes the water activity coefficient, and $\Phi_{0}$ represents the initial solute volume fraction in the droplet, which is primarily determined by the droplet’s origin. Respiratory activities result in different solute mass fractions, as shown in Table 1. Due to higher concentrations of respiratory tract lining fluid in the oral cavity, droplets generated here typically have a $\Phi_{0}$ value of approximately 1–2% [43]. In contrast, droplets produced deeper in the respiratory tract by coughing or sneezing often exhibit $\Phi_{0}$ values between 2 and 3% [40]. Droplets originating from the lungs, influenced by alveolar surfactant, generally have lower concentrations, around 0.5–1% [24]. Larger residual nuclei from evaporating droplets tend to have higher solute mass fractions due to their extended air suspension time. In cases of respiratory infection or inflammation, increased protein concentrations in the airway can further elevate $\Phi_{0}$. For modeling purposes, an initial nonvolatile solute mass fraction of 2% is commonly adopted and is used in the subsequent simulations with the Wells evaporation model to estimate the final droplet-nucleus size.

In practice, the radius of droplets may not fully evaporate to the residual radius $R_{\mathrm{ev}}$ before settling. Therefore, the relationship between sedimentation time and evaporation time must be considered. Sedimentation time $t_{\mathrm{sed}}$ is the duration from emission until droplets settle on a surface, described via the vertical velocity as $h=\int_{0}^{t_{\mathrm{sed}}}v_{z}\left(t\right)\;dt$, where *h* is the emission height. The evaporation time $t_{\mathrm{ev}}$ is the period required to reach $R_{\mathrm{ev}}$; setting $R\left(t\right)=R_{\mathrm{ev}}$ in Equation (1) gives $t_{\mathrm{ev}}=R_{0}^{2}\left[1-R_{\mathrm{ev}}^{2}/R_{0}^{2}\right]/\left[\theta \left(1-\mathrm{RH}\right)\right]$, which is influenced by $R_{0}$, RH, and solute properties [44].

The evaporation and sedimentation times are equal at $R_{0}=42$ μm for RH = 0.25, $R_{0}=49$ μm for RH = 0.50, and $R_{0}=54$ μm for RH = 0.75. When $R_{0}$ is smaller than these critical values, droplets evaporate completely before settling; for larger droplets, sedimentation occurs first, leading to longer airborne durations.

### 2.2. Different Respiratory Activities Have Different Droplet Sizes

Human respiratory activities such as coughing, speaking, and sneezing release droplets spanning a wide size spectrum, which have been identified as potential carriers of SARS-CoV-2 [1,45,46,47]. Initial droplet velocity, number concentration, and size distribution vary substantially among breathing, speaking, coughing, and sneezing, as summarized in Table 2 [18,19,20].

During quiet breathing, respiratory flow rates average 19.55 L/min (21.0 L/min for males and 18.055 L/min for females) [49]. The oral cross-sectional area during exhalation typically measures 2.5 cm^2^, ranging between 1.5 and 4 cm^2^—approximately 1.5–2 times larger than the laryngeal opening [50]. Applying the continuity equation, the corresponding airflow velocity ranges from 0.81 to 2.17 m/s, with a mean of 1.30 m/s. Breathing predominantly generates droplets in the 0.1–5 μm range, with a mode radius of 0.8 μm [40].

Speaking produces droplets with an initial velocity of 5 m/s, sizes ranging from 0.5 to 50 μm, and a mode radius of 1.6 μm [40]. Coughing exhibits higher initial velocities (10–20 m/s) and a broader bimodal size distribution (0.5–500 μm), with peaks at 1.5 μm and 74 μm, reflecting the simultaneous emission of fine aerosols and larger droplets [37].

Sneezing, characterized by even higher initial velocities (30–100 m/s) due to distinct biomechanics, also demonstrates a bimodal droplet distribution, with modes at 74–86 μm and 340–400 μm [18,19,20,48], indicating substantial emission of both small and large droplets.

In this study, each emission event is modeled as a horizontal projection. The initial velocities listed in Table 2 are assigned as $v_{0}$ in the x direction, with zero initial velocity in the z direction. The modal radius of each activity is adopted as the characteristic droplet size for subsequent modeling. Measured droplet sizes are influenced not only by respiratory effort but also by experimental methodology and the health status of the subjects. For instance, sampling location relative to droplet formation may introduce evaporation-related artifacts. Such factors are difficult to quantify systematically and are not explicitly addressed herein.

**Figure 3:**
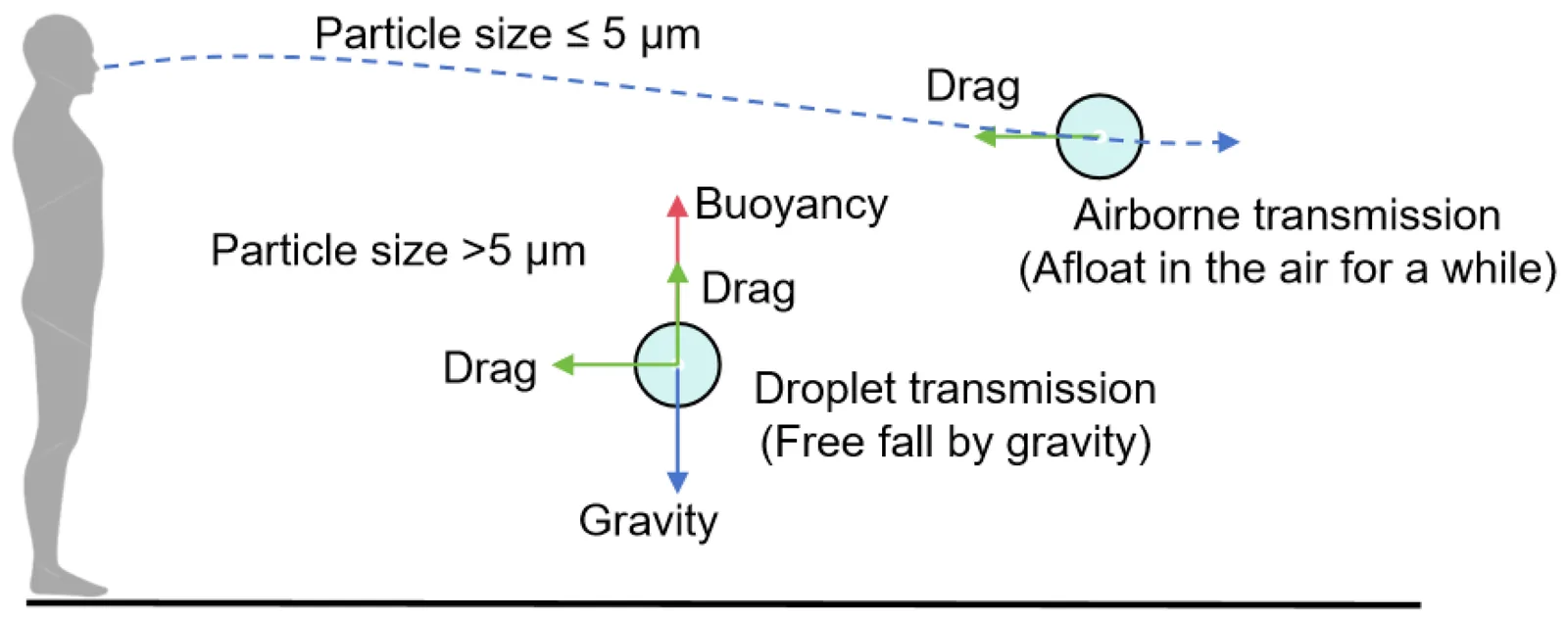
Schematic diagram illustrating the classification of droplet transmission and aerosol transmission, with the boundary distinguishing the dominant forces acting on droplets.

It should be noted that the smallest droplet sizes considered in this study (0.1–0.5 µm in Table 2) correspond to the lower detection limits of conventional aerosol instruments; individual virions, with characteristic sizes on the order of 50–150 nm, are not resolved. In practice, respiratory droplets act as carriers for virus particles, and the collective transport behavior of the droplet–virus aggregate is governed by the droplet’s aerodynamic properties rather than the virion size alone [46]. In the medical literature, “droplet” and “aerosol” are often used interchangeably. The concept of a critical size, introduced by Wells in 1934, provides a physical basis for distinguishing transmission routes. Under dry conditions, droplets below this threshold can fully evaporate into droplet nuclei that remain airborne and enable long-range transmission. In humid environments, evaporation slows, inhibiting rapid settling due to reduced particle mass. Following Wells’ theory and WHO guidelines, a diameter of 5 µm (equivalent to a radius of 2.5 µm) serves as the conventional boundary between droplets and aerosols. Particles larger than this threshold (radius >2.5 µm) are predominantly affected by gravity, resulting in rapid sedimentation and short-range transmission. Those at or below this threshold (radius ≤2.5 µm) readily form droplet nuclei that remain suspended for extended periods, facilitating long-range aerosol transmission. Figure 3 illustrates the radius-dependent trajectory variation [24]. Accordingly, this study models droplets with radii >2.5 µm by emphasizing gravitational sedimentation, whereas particles with radii ≤2.5 µm are modeled primarily in terms of Brownian motion, with gravitational effects neglected.

### 2.3. Dynamic Modeling of Droplets

This section derives the velocity equations for droplets in the x and y directions under different force conditions. The drag force acting on the droplets varies with both their velocity and radius, and the Reynolds number is employed to characterize the resistance encountered during transmission. The Reynolds number for droplets is defined as

(3) Rep=2ρav(t)R(t)η,

where R(t) represents the radius of the droplet at time *t*, $\rho_{a}$ is the air density, and η is the dynamic viscosity of air ($\eta \approx 1.85\times 10^{-5}\;\mathrm{kg}/\left(m\cdot s\right)$). As droplets travel through the air, their motion in the x and z directions is governed primarily by gravity, buoyancy, and drag, whereas their motion in the y direction is mainly influenced by Brownian motion. The velocities in the x and z directions are denoted as $v_{x}\left(t\right)$ ($v_{x}\left(t\right)>0$) and $v_{z}\left(t\right)$ ($v_{z}\left(t\right)<0$), respectively [37]. The Reynolds number for the instantaneous velocity is given by Rep=2ρavx2(t)+vz2(t)R(t)η. For relatively large Reynolds numbers (Rep>0.1), the drag force acting on the droplets is dominated by inertial effects [51]. The forces acting on the droplets in the x and z directions can be expressed as

(4) $$\begin{array}{c} F_{x}\left(t\right)=-\frac{1}{2}\rho_{a}Kv_{x}^{2}\left(t\right)\pi R^{2}\left(t\right), \end{array}$$

(5) $$\begin{array}{c} F_{z}\left(t\right)=\frac{4}{3}\pi R^{3}\left(t\right)g\left(\rho_{a}-\rho_{d}\right)-\frac{1}{2}\rho_{a}Kv_{z}\left(t\right)\left|v_{z}\left(t\right)\right|\pi R^{2}\left(t\right), \end{array}$$

where *K* is the drag coefficient, and *g* is the acceleration due to gravity. The sign convention defines the *z*-axis as positive upward, such that $v_{z}\left(t\right)<0$ corresponds to downward settling, and the drag force opposes the instantaneous velocity throughout. The evaporation process leads to changes in droplet radius, which in turn directly affects droplet mass. Therefore, the droplet mass *m* can be represented as $m=\frac{4}{3}\pi \rho_{d}R^{3}\left(t\right)$, where $R\left(t\right)=\sqrt{R_{0}^{2}-t\theta \left(1-\mathrm{RH}\right)}$. Substituting these into the velocity expressions for $v_{x}\left(t\right)$ and $v_{z}\left(t\right)$ gives as

(6) $$\begin{array}{c} v_{x}\left(t\right)=\frac{1}{\frac{3\rho_{a}K}{4\rho_{d}\theta \left(1-\mathrm{RH}\right)}\left(R_{0}-\sqrt{R_{0}^{2}-t\theta \left(1-\mathrm{RH}\right)}\right)+\frac{1}{v_{0}}}, \end{array}$$

(7) $$\begin{array}{c} v_{z}\left(t\right)=\sqrt{\frac{8g\left(\rho_{a}-\rho_{d}\right)R_{0}}{3\rho_{a}K}}\;\tanh\left(\sqrt{\frac{3g\left(\rho_{a}-\rho_{d}\right)\rho_{a}K}{8\rho_{d}^{2}R_{0}}}\;t\right). \end{array}$$

Due to evaporation and drag, both the droplet radius and velocity decrease continuously over time, leading to a corresponding decrease in the Reynolds number (Rep). When Rep≤0.1, the force acting on the droplet transitions to the Stokes regime. For analytical tractability, the drag formulation is piecewise: the preceding equations describe the inertial regime and the following equations the Stokes regime; a single continuous drag coefficient valid across all Reynolds numbers is provided by the Morrison correlation [52]. The forces along the x and z directions can be expressed as

(8) $$\begin{array}{cc} F_{x}\left(t\right) & =-6\pi \eta R\left(t\right)v_{x}\left(t\right), \end{array}$$

(9) $$\begin{array}{cc} F_{z}\left(t\right) & =\frac{4}{3}\pi R^{3}\left(t\right)g\left(\rho_{a}-\rho_{d}\right)+6\pi \eta R\left(t\right)v_{z}\left(t\right). \end{array}$$

Here, the drag force $6\pi \eta Rv_{z}$ scales linearly with the droplet radius, whereas the gravitational force scales with its cube. Therefore, when the droplet radius is halved, the drag force is reduced by half, whereas the gravitational force decreases by a factor of eight. As a result, smaller droplets can remain suspended in the air for a longer duration. The expressions for $v_{x}\left(t\right)$ and $v_{z}\left(t\right)$ can be derived as

(10) $$\begin{array}{c} v_{x}\left(t\right)=v_{0}{\left(\frac{R_{0}^{2}-t\theta \left(1-\mathrm{RH}\right)}{R_{0}^{2}}\right)}^{\frac{9\eta }{2\rho_{d}\theta \left(1-\mathrm{RH}\right)}}, \end{array}$$

(11) $$\begin{array}{cc} v_{z}\left(t\right) & =\frac{2g(\rho_{a}-\rho_{d})}{2\rho_{a}\theta \left(1-\mathrm{RH}\right)+9\eta }\\ \; & \times \left(R_{0}^{2}{\left(\frac{R_{0}^{2}}{R_{0}^{2}-t\theta \left(1-\mathrm{RH}\right)}\right)}^{\frac{9\eta }{2\rho_{d}\theta \left(1-\mathrm{RH}\right)}}-\left(R_{0}^{2}-t\theta \left(1-\mathrm{RH}\right)\right)\right) \end{array}.$$

The above formulation applies when droplets settle before reaching their residual radii, i.e., when $t_{\mathrm{sed}}\leq t_{\mathrm{ev}}$. In this case, the droplet radius used in $v_{x}\left(t\right)$ and $v_{z}\left(t\right)$ is denoted as R(t). For $t_{\mathrm{sed}}>t_{\mathrm{ev}}$, the droplet radius has reached $R_{\mathrm{ev}}$ before settling. Therefore, $t\leq t_{\mathrm{ev}}$, the droplet radius in $v_{x}\left(t\right)$ and $v_{z}\left(t\right)$ is R(t), while for $t_{\mathrm{ev}}<t\leq t_{\mathrm{sed}}$, the radius is $R_{\mathrm{ev}}$. For example, for droplets with a radius greater than 2.5 µm and Rep>0.1, the force in the x direction can be expressed as

(12) $$\begin{array}{c} F_{x}\left(t\right)=-\frac{1}{2}\rho_{a}Kv_{x}\left(t\right)R^{2}\left(t\right),\;\left(t_{\mathrm{sed}}\leq t_{\mathrm{ev}}\right) \end{array}$$

(13) $$\begin{array}{c} F_{x}\left(t\right)=-\frac{1}{2}\rho_{a}Kv_{x}\left(t\right)R^{2}\left(t\right),\;\left(t_{\mathrm{sed}}>t_{\mathrm{ev}},\;0<t\leq t_{\mathrm{ev}}\right) \end{array}$$

(14) $$\begin{array}{c} F_{x}\left(t\right)=-\frac{1}{2}\rho_{a}Kv_{x}\left(t\right)R_{\mathrm{ev}}^{2}.\;\left(t_{\mathrm{sed}}>t_{\mathrm{ev}},\;t_{\mathrm{ev}}<t\leq t_{\mathrm{sed}}\right) \end{array}$$

For droplet radii less than or equal to 2.5 µm (R≤2.5 µm), the gravitational settling velocity becomes negligible compared to Brownian diffusion and advective transport. Based on the Stokes–Einstein relation, the terminal settling velocity scales approximately as $R^{2}$; for R≤2.5μm, the terminal velocity is on the order of $3\times 10^{-4}\;m/s$, which is at least two orders of magnitude smaller than typical indoor airflow velocities [53]. Therefore, gravity can be neglected, i.e., $F_{z}\left(t\right)=0$, $v_{z}\left(t\right)=0$. For droplets that were initially larger but have evaporated to R≤2.5μm before settling, their residual vertical velocity is approximately equal to the terminal velocity corresponding to the equilibrium radius $R_{\mathrm{ev}}$, which remains negligibly small. The velocity and force in the x direction are derived as described earlier. A detailed derivation of the velocity equations in the x and z directions is provided in Appendix A.

**Figure 4:**
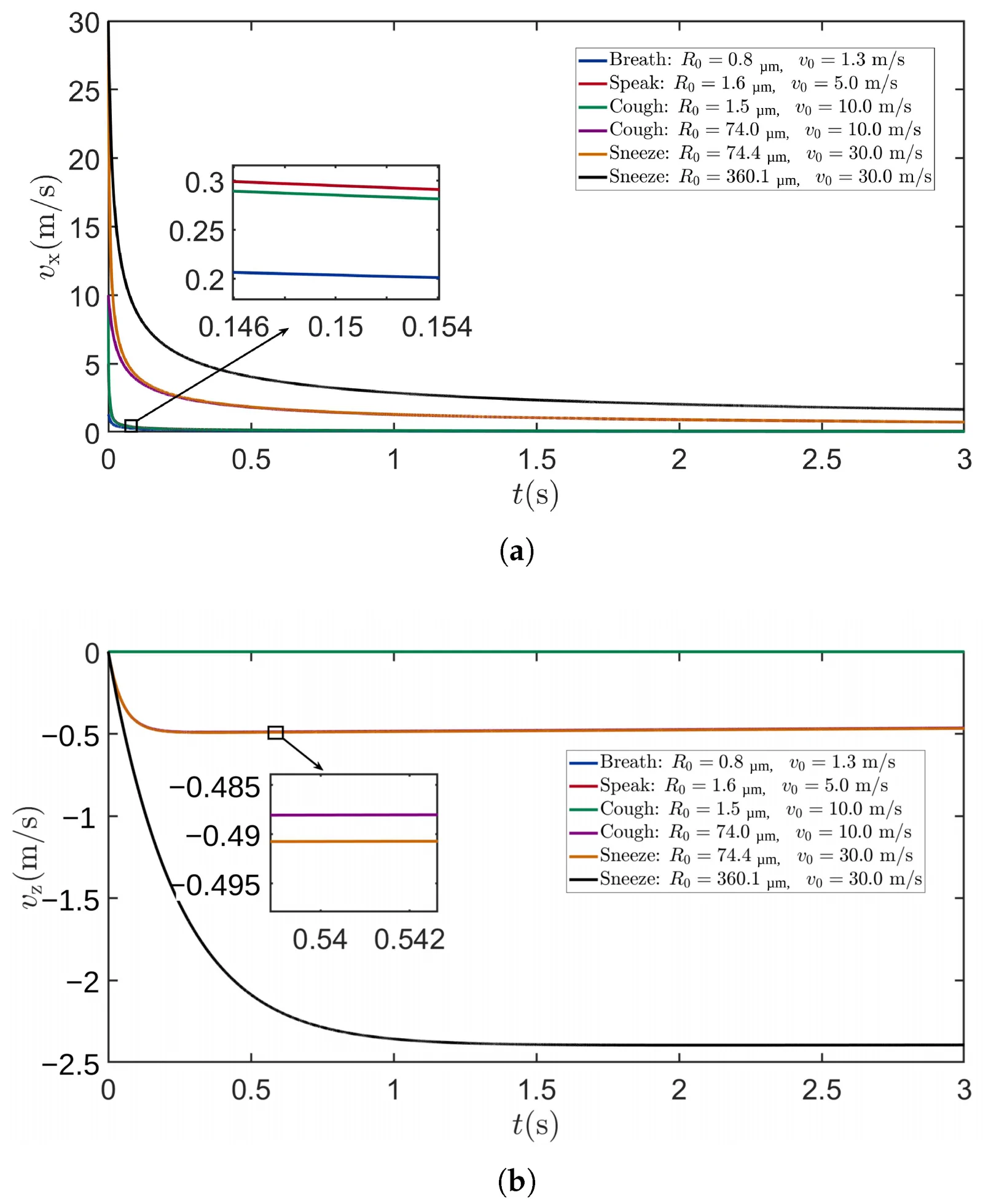
RH = 0.5. For different respiratory modes, (**a**) the relationship between time *t* and velocity $v_{x}\left(t\right)$; (**b**) the relationship between time *t* and velocity $v_{z}\left(t\right)$.

Figure 4 illustrates the relationship between droplet velocities $v_{x}$ and $v_{z}$ for different respiratory modes. As presented in Figure 4a, droplet velocity decreases rapidly within a very short time in all cases. The steeper the curve, the larger the initial velocity, as observed in coughing and sneezing, due to the inverse relationship between air resistance and velocity. As presented in Figure 4b, for small droplets during breathing, speaking, and coughing, gravity’s influence is negligible due to their smaller size, while the other curves exhibit a rapid decline under the influence of gravity. Additionally, the maximum velocity for larger droplets during sneezing is significantly higher than in other scenarios, reaching up to 2.4 m/s, which is substantially greater than the 0.5 m/s observed for droplets with radii of 74 µm and 74.4 µm. This is because larger droplets have a smaller surface area-to-mass ratio, leading to reduced deceleration from air resistance compared to smaller droplets, thus enabling them to settle more easily under gravity. As the forces of drag and buoyancy continue to act, the acceleration decreases further. Eventually, when the forces of buoyancy, gravity, and drag reach equilibrium, the velocity curves stabilize.

The displacements of droplets in the x and z directions, $s_{x}\left(t\right)$ and $s_{z}\left(t\right)$, can be obtained by integrating the above velocity equations $v_{x}\left(t\right)$ and $v_{z}\left(t\right)$ to yield [36]

(15) $$\begin{array}{c} s_{x}\left(t\right)=\int_{0}^{t}v_{x}\left(\tau \right)\;d\tau , \end{array}$$

(16) $$\begin{array}{c} s_{z}\left(t\right)=\int_{0}^{t}v_{z}\left(\tau \right)\;d\tau . \end{array}$$

**Figure 5:**
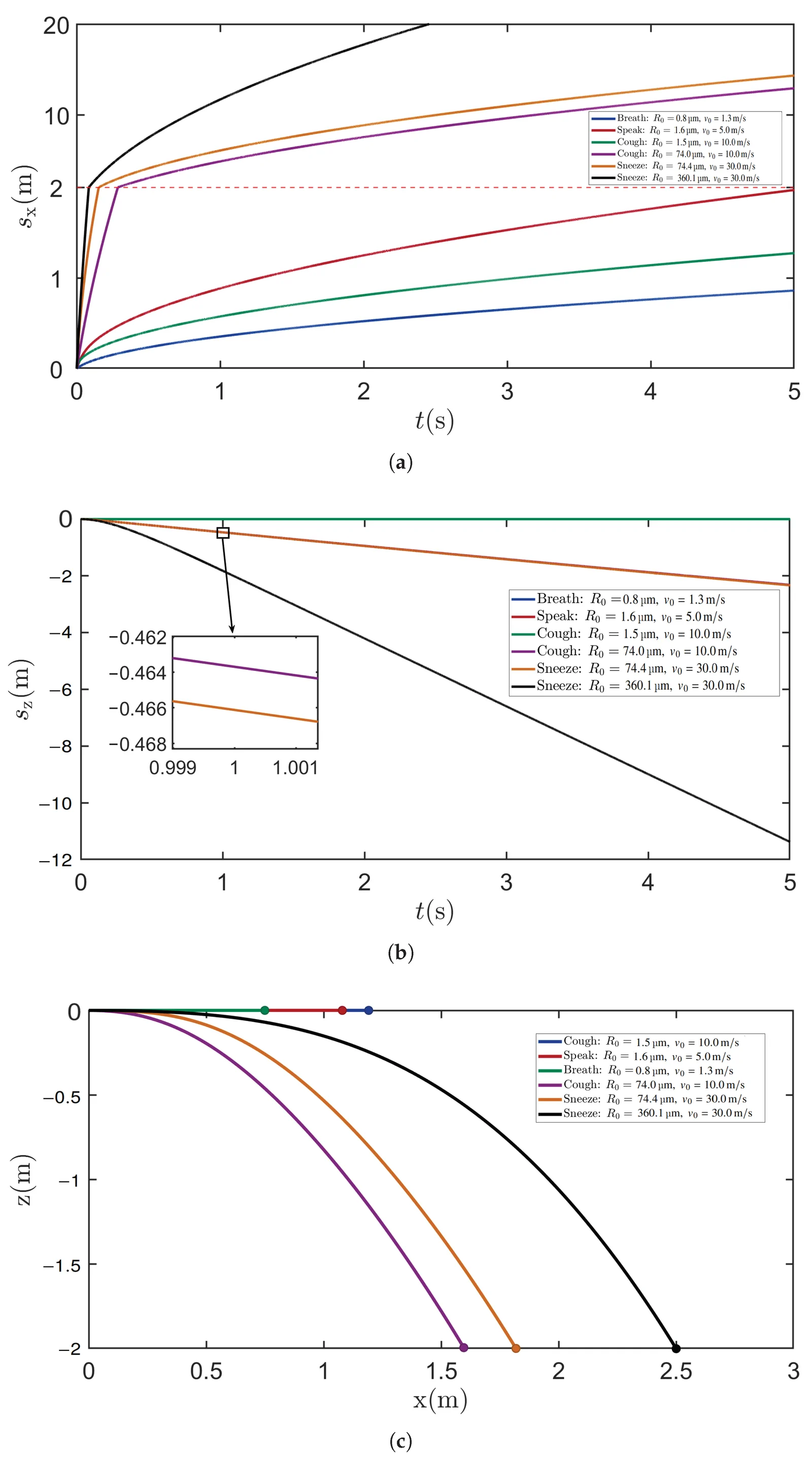
At RH = 0.5, under different respiratory modes: (**a**) the relationship between time t and displacement; (**b**) the relationship between time t and displacement; (**c**) the propagation trajectories of droplets.

The three panels in Figure 5 show the $s_{x}$ and $s_{z}$ displacement curves and the propagation trajectories of droplets generated by different respiratory activities, including breathing, speaking, coughing, and sneezing. Figure 5a shows that the horizontal displacement of droplets increases significantly with both initial velocity ($v_{0}$) and initial radius ($R_{0}$). Droplets generated by sneezing can travel horizontally over 20 m within 5 s without settling, while droplets from breathing only displace about 1 m. Although the velocity during coughing is greater than that during speaking, droplets with a radius of 1.5 µm from coughing exhibit a horizontal displacement similar to that of droplets from speech, highlighting the critical role of droplet size in determining propagation behavior. As presented in Figure 5b, the vertical displacement curve reveals that droplets with a radius smaller than 2.5 µm remain stationary at 0, while larger droplets exhibit the same trend observed in Figure 5a: increased size results in greater descending speed and distance. Figure 5c integrates the two-dimensional trajectories of $s_{x}$ and $s_{z}$, showing that despite their higher horizontal velocity, large droplets quickly settle to the ground due to their vertical motion, which limits their horizontal propagation distance.

These results indicate that particle size is a critical factor in determining propagation behavior. Smaller particles can remain suspended for extended periods and travel over significant distances, while larger particles tend to settle more quickly. The results further demonstrate that high initial velocities ($v_{0}>$ 20 m/s) can substantially increase effective transmission distances and generate turbulent wakes. Notably, particles between 1 and 10 µm are most susceptible to air flow disturbances, which explains why aerosols generated during speech exhibit a higher-than-expected transmission capacity. These findings suggest that the current recommendation of a 2 m social distance is effective under normal conditions. However, during events involving powerful coughs or the expulsion of large droplets, this distance proves inadequate and should be increased to over 2.5 m. These distance estimates are based on the idealized quiescent-air model and should be interpreted as indicative upper bounds; in practice, room-specific airflow patterns, occupant movement, and furniture layout can substantially alter actual transmission distances [27]. For even finer particulate sizes, N95-level protection is necessary [54,55].

### 2.4. Airborne Concentration

The airborne concentration distributions of droplets vary substantially among respiratory activities, such as quiet breathing, speaking, coughing, and sneezing, owing to differences in flow characteristics (e.g., velocity and turbulence intensity) and initial emission conditions (e.g., droplet size distribution and initial jet velocity). Thus, the concentration distribution in the air is not uniform. First, the movement of the liquid in flow can exhibit different flow states, such as laminar or turbulent flow. Second, there are substantial differences in mixing ability and diffusive characteristics based on the velocity. To determine the flow state of the gas, the Reynolds number can be assessed as

(17) Ref=ρav0Lη,

where $\rho_{a}$ is the density of the exhaled air, approximately 1.2 ${\mathrm{kg}/m}^{3}$, $v_{0}$ is the initial velocity as the air leaves the mouth, *L* is the characteristic radius of the mouth opening, approximately 2cm, and η is the dynamic viscosity of air, approximately $1.8\times 10^{-5}\;\mathrm{Pa}\cdot s$. When Ref is approximately 2000, the flow usually exhibits laminar characteristics, indicating orderly flow lines and motion; when Ref exceeds about 4000, the flow typically becomes turbulent, particularly showing strong vortices and irregular velocities. The flow exists in a transitional state between laminar and turbulent flow when Ref is between 2000 and 4000. By substituting the data from Table 2, we obtain Ref for different respiratory activities. The average velocity during quiet exhalation is approximately 1.3 m/s, corresponding to Ref≈1.7×103, which lies within the laminar regime. During speaking, the velocity increases to 5 m/s, resulting in Ref≈6.7×103, with the jet becoming fully turbulent at a distance of 2 to 3 radii from the mouth. The velocity during coughing ranges from 10 to 20 m/s, achieving Ref values between $1.3\times 10^{4}$ and $2.7\times 10^{4}$, with the expelled air exhibiting typical turbulence immediately at the outlet. The velocity of a sneeze can reach 30 to 100 m/s, further increasing the Ref to between $4.0\times 10^{4}$ and $1.3\times 10^{5}$, forming a strong turbulent puff cloud. The following analysis examines different respiratory activities separately to quantify their respective transport characteristics.

For breathing and speaking, the normal respiratory rate of adult individuals ranges from 12 to 16 breaths per minute, meaning each breath lasts up to 4.98 s. Since each exhalation is brief relative to the overall experimental duration, fluctuations in droplet emission caused by individual exhalations can be neglected. For sustained speech (such as reciting a passage or engaging in continuous conversation), this process can be approximated as a continuous and uniform emission over a certain duration. Thus, human breathing and speaking can be treated as continuous and steady emission processes, with differences in the quantity and velocity of droplets emitted between the two activities, as shown in Table 2. Assuming that a person enters a room and remains there long enough for the system to reach a detectable steady state, only the steady-state response needs to be considered. Since the steady-state response is time-independent, the aerosol concentration in the system no longer depends on time but is primarily characterized by its spatial distribution. However, while the system reaches a steady state, the velocity of aerosols during propagation is not constant; it gradually decreases over time due to environmental resistance. Additionally, the modal radii of droplets produced during exhalation and speaking are below the critical diameter of 5 µm in diameter (radius 2.5 µm); therefore, gravitational effects on these droplets are negligible, allowing us to focus solely on their velocity in the x direction, $v_{x}\left(t\right)$. To accurately characterize the non-constant jet velocity under steady-state conditions, the originally time-dependent $v_{x}\left(t\right)$ must be converted into a spatial position function, $v_{x}\left(x\right)$. Specifically, by analyzing the evolution of a single droplet in space, the time-dependent velocity relationship can be mapped to a position-dependent one. Given the time-dependent velocity function $v_{x}\left(t\right)$ and based on $s_{x}\left(t\right)$, the relationship can be derived [36] as

(18) sx(t)=∫0tv0exp−3ρaK4ρdθ(1−RH)R0−R02−τθ(1−RH)dτ,Rep>0.1,∫0tv0R02−τθ(1−RH)R029η2ρdθ(1−RH)dτ,Rep≤0.1.

Here, the displacement $s_{x}\left(t\right)$ represents the position x, and thus the correspondence between time and space, t(x), can be obtained through the inverse function. However, this integral is generally challenging to solve analytically using elementary functions. Therefore, the relationship can be expressed using the inverse function notation as

(19) $$\begin{array}{c} t\left(x\right)=s_{x}^{-1}\left(x\right). \end{array}$$

Substituting t(x) into the time-dependent velocity $v_{x}\left(t\right)$ yields the following position-dependent velocity

(20) $$\begin{array}{c} v_{x}\left(x\right)=v_{x}\left(t\left(x\right)\right)=v_{x}\left(s_{x}^{-1}\left(x\right)\right). \end{array}$$

Next, using the fundamental fluid dynamics framework, the concentration function is derived. Applying the law of mass conservation, the divergence of the mass flux equals the source term [37], yielding

(21) $$\begin{array}{c} \nabla \cdot \vec{F}=S\left(x,y,z\right), \end{array}$$

where S(x,y,z) represents the molecular source term, and $\vec{F}$ denotes the mass flux, comprising both the diffusive component, ${\vec{F}}_{\mathrm{diff}}$, and the advective component, ${\vec{F}}_{\mathrm{adv}}$. Note that the divergence operator ∇· (rather than the gradient ∇) is used here to represent mass conservation. The derivation methods for this equation vary depending on the specific environment. Here, the focus is on the flux terms, which incorporate Fickian diffusion and advective transport to model diffusion and transient flow [36]. The relationship is given by

(22) $$\begin{array}{c} \vec{F}={\vec{F}}_{\mathrm{diff}}+{\vec{F}}_{\mathrm{adv}}. \end{array}$$

In both breathing and speaking scenarios, the velocity component along the z direction is zero, meaning advective flow occurs solely along the x direction. Consequently, the divergence of the advective term ${\vec{F}}_{\mathrm{adv}}$ is

(23) $$\begin{array}{c} \nabla \cdot {\vec{F}}_{\mathrm{adv}}=v_{x}\left(x\right)\frac{\partial C(x,y,z)}{\partial x}. \end{array}$$

Along the direction of advection, advective transport dominates diffusion; therefore, the diffusive flux in the x direction can be neglected. Accordingly, only the y- and z-components of the diffusive flux are considered, and the divergence of ${\vec{F}}_{\mathrm{diff}}$ is given by

(24) $$\begin{array}{c} \nabla \cdot {\vec{F}}_{\mathrm{diff}}=-D\left(\frac{\partial^{2}C\left(x,y,z\right)}{\partial y^{2}}+\frac{\partial^{2}C\left(x,y,z\right)}{\partial z^{2}}\right), \end{array}$$

where *D* represents the turbulent diffusion coefficient, quantifying the extent of turbulent diffusion. For analytical simplicity, *D* is assumed to be constant throughout this study. It should be emphasized that this gradient-diffusion parameterization describes the ensemble-averaged spreading of the droplet cloud rather than the Lagrangian entrainment of individual droplets by resolved turbulent eddies; the implications of this distinction for the comparability of the model with CFD-resolved airflow fields are discussed in Section 5. Combining the advective and diffusive flux divergences yields the divergence of the total mass flux

(25) $$\begin{array}{c} \nabla \cdot \vec{F}=v_{x}\left(x\right)\frac{\partial C(x,y,z)}{\partial x}-D\left(\frac{\partial^{2}C\left(x,y,z\right)}{\partial y^{2}}+\frac{\partial^{2}C\left(x,y,z\right)}{\partial z^{2}}\right). \end{array}$$

If the position of a person’s mouth is at the coordinates [0,0,h], the source term can be expressed as

(26) $$\begin{array}{c} S\left(x,y,z\right)=Nv_{x}\left(x\right)C\left(0,0,z\right)\delta \left(y\right)\delta \left(z-h\right), \end{array}$$

where *N* denotes the total number of aerosol particles released during a single respiratory event. It should be noted that the airflow during human exhalation is in the laminar regime, which implies that the diffusion coefficient relevant to breathing differs from the turbulent diffusion coefficient characteristic of the fully turbulent layer associated with speaking. To characterize diffusion in these two distinct flow regimes, two parameters are introduced: the diffusion coefficient in the laminar region, $D_{\mathrm{tr}}$, and the turbulent diffusion coefficient in the turbulent region, $D_{\mathrm{tu}}$. The concentration during breathing is denoted by $C_{\mathrm{breath}}$, whereas the concentration during speaking is denoted by $C_{\mathrm{speak}}$. The total number of droplets emitted during these two activities is represented by $N_{\mathrm{breath}}$ and $N_{\mathrm{speak}}$, respectively. The concentration during breathing is given by

(27) $$\begin{array}{ccc} C_{\mathrm{breath}}\left(x,y,z\right) & = & \frac{N_{\mathrm{breath}}}{4\pi D_{\mathrm{tr}}v_{x}\left(x\right)}\exp\left(-\frac{y^{2}}{4D_{\mathrm{tr}}\int_{0}^{x}\frac{1}{v_{x}\left(x^{\prime }\right)}dx^{\prime }}\right)\\ & & \times \left(\exp\left(-\frac{{\left(z-h\right)}^{2}}{4D_{\mathrm{tr}}\int_{0}^{x}\frac{1}{v_{x}\left(x^{\prime }\right)}dx^{\prime }}\right)+\exp\left(-\frac{{\left(z+h\right)}^{2}}{4D_{\mathrm{tr}}\int_{0}^{x}\frac{1}{v_{x}\left(x^{\prime }\right)}dx^{\prime }}\right)\right).\;\;\;\;\;\;\;\;\;\; \end{array}$$

The concentration for speaking, denoted as $C_{\mathrm{speak}}$, is analogously expressed as

(28) $$\begin{array}{c} \begin{array}{cc} C_{\mathrm{speak}}\left(x,y,z\right) & =\frac{N_{\mathrm{speak}}}{4\pi D_{\mathrm{tu}}v_{x}\left(x\right)}\exp\left(-\frac{y^{2}}{4D_{\mathrm{tu}}\int_{0}^{x}\frac{1}{v_{x}\left(x^{\prime }\right)}dx^{\prime }}\right)\\ & \times \left(\exp\left(-\frac{{\left(z-h\right)}^{2}}{4D_{\mathrm{tu}}\int_{0}^{x}\frac{1}{v_{x}\left(x^{\prime }\right)}dx^{\prime }}\right)+\exp\left(-\frac{{\left(z+h\right)}^{2}}{4D_{\mathrm{tu}}\int_{0}^{x}\frac{1}{v_{x}\left(x^{\prime }\right)}dx^{\prime }}\right)\right). \end{array} \end{array}$$

Unlike breathing and speaking, coughing and sneezing are characterized by impulsive aerosol emissions. A single cough or sneeze is modeled as an instantaneous point source that releases a specified number of droplets into the air at t=0. Similarly, assuming the initial source is located at coordinates [0,0,h], the corresponding source term can be modeled as

(29) $$\begin{array}{c} S(x,y,z,t)=N\delta (x)\delta (z-h)\delta (t). \end{array}$$

For human coughing, according to the data in Table 2, droplet radii have two typical values, with radii of 1.5 µm and 74 µm accounting for the majority of droplets. For droplets with a radius of 74 µm, based on mass conservation [44,45], the governing equation is

(30) $$\begin{array}{c} \frac{\partial C(x,y,z,t)}{\partial t}+\nabla \cdot \vec{F}=S\left(x,y,z,t\right). \end{array}$$

Here, $\frac{\partial C(x,y,z,t)}{\partial t}$ denotes the change in particle concentration with time, and S(x,y,z,t) is the initial molecular source term representing the release from the infectious source. Similarly, $\vec{F}$ is composed of ${\vec{F}}_{\mathrm{diff}}$ and ${\vec{F}}_{\mathrm{adv}}$, but for large droplets that exceed the critical settling size, the velocity induced by gravity in both the x and z directions must be considered. Thus, the divergence of the advective term is

(31) $$\begin{array}{c} \nabla \cdot {\vec{F}}_{\mathrm{adv}}=v_{x}\left(x\right)\frac{\partial C(x,y,z,t)}{\partial x}+v_{z}\left(z\right)\frac{\partial C(x,y,z,t)}{\partial z}. \end{array}$$

Along the flow directions, advective transport dominates diffusion; therefore, the diffusive fluxes in the x and z directions can be neglected. Therefore, only the diffusive flux in the y direction is considered. Thus, the divergence of the diffusive flux is

(32) $$\begin{array}{c} \nabla \cdot {\vec{F}}_{\mathrm{diff}}=-D_{\mathrm{tu}}\left(\frac{\partial^{2}C\left(x,y,z,t\right)}{\partial y^{2}}\right). \end{array}$$

Coughing and sneezing both correspond to the fully turbulent flow regime, where the diffusion coefficient is $D_{\mathrm{tu}}$. The resulting partial differential equation is given by

(33) $$\begin{array}{c} \frac{\partial C(x,y,z,t)}{\partial t}+v_{x}\left(t\right)\frac{\partial C(x,y,z,t)}{\partial x}+v_{z}\left(t\right)\frac{\partial C(x,y,z,t)}{\partial z}-D_{\mathrm{tu}}\left(\frac{\partial^{2}C\left(x,y,z,t\right)}{\partial y^{2}}\right)\\ =N\delta (x)\delta (y)\delta (z-h)\delta (t).\; \end{array}$$

For *N*, the ratio of particle counts with radii of 1.5 µm and 74 µm is 5.6:1 [46]. Based on the partial differential equation, the concentration of 74 µm droplets during coughing is [25]

(34) $$\begin{array}{ccc} C_{\mathrm{cough}}\left(x,y,z,t\right) & = & \frac{10N_{\mathrm{cough}}}{66{\left(4\pi D_{\mathrm{tu}}t\right)}^{3/2}}\exp\left(-\frac{{\left(x-s_{x}\left(t\right)\right)}^{2}+y^{2}}{4D_{\mathrm{tu}}t}\right)\\ & & \times \left(\exp\left(-\frac{{\left(z-h-s_{z}\left(t\right)\right)}^{2}}{4D_{\mathrm{tu}}t}\right)+\exp\left(-\frac{{\left(z+h-s_{z}\left(t\right)\right)}^{2}}{4D_{\mathrm{tu}}t}\right)\right).\;\;\;\;\;\; \end{array}$$

For droplets with a radius of 1.5 µm, the velocity component in the z direction is negligible compared with that of droplets with a radius of 74 µm. Therefore, the corresponding concentration is

(35) $$\begin{array}{ccc} C_{\mathrm{cough}}\left(x,y,z,t\right) & = & \frac{56N_{\mathrm{cough}}}{66{\left(4\pi D_{\mathrm{tu}}t\right)}^{3/2}}\exp\left(-\frac{{\left(x-s_{x}\left(t\right)\right)}^{2}+y^{2}}{4D_{\mathrm{tu}}t}\right)\\ & & \times \left(\exp\left(-\frac{{\left(z-h\right)}^{2}}{4D_{\mathrm{tu}}t}\right)+\exp\left(-\frac{{\left(z+h\right)}^{2}}{4D_{\mathrm{tu}}t}\right)\right). \end{array}$$

For sneezing, Table 2 shows two modal droplet radii: 74.4 µm and 360.1 µm. Based on high-speed imaging data, these two radii account for approximately 68% and 22% of the collected droplets, respectively [48]. Since both radii are substantially larger than the critical radius of 2.5 µm (diameter: 5 µm), the initial velocity and position in the z direction must be considered in both cases. The derivation follows the same process as for the 74 µm droplets in coughing, with the only difference being the droplet radius and initial number. Therefore, for droplets with a 74.4 µm radius, the concentration is

(36) $$\begin{array}{ccc} C_{\mathrm{sneeze}}\left(x,y,z,t\right) & = & \frac{68\%N_{\mathrm{sneeze}}}{{\left(4\pi D_{\mathrm{tu}}t\right)}^{3/2}}\exp\left(-\frac{{\left(x-s_{x}\left(t\right)\right)}^{2}+y^{2}}{4D_{\mathrm{tu}}t}\right)\\ & & \times \left(\exp\left(-\frac{{\left(z-h-s_{z}\left(t\right)\right)}^{2}}{4D_{\mathrm{tu}}t}\right)+\exp\left(-\frac{{\left(z+h-s_{z}\left(t\right)\right)}^{2}}{4D_{\mathrm{tu}}t}\right)\right). \end{array}$$

The concentration for droplets with a 360.1 µm radius is

(37) $$\begin{array}{ccc} C_{\mathrm{sneeze}}\left(x,y,z,t\right) & = & \frac{22\%N_{\mathrm{sneeze}}}{{\left(4\pi D_{\mathrm{tu}}t\right)}^{3/2}}\exp\left(-\frac{{\left(x-s_{x}\left(t\right)\right)}^{2}+y^{2}}{4D_{\mathrm{tu}}t}\right)\\ & & \times \left(\exp\left(-\frac{{\left(z-h-s_{z}\left(t\right)\right)}^{2}}{4D_{\mathrm{tu}}t}\right)+\exp\left(-\frac{{\left(z+h-s_{z}\left(t\right)\right)}^{2}}{4D_{\mathrm{tu}}t}\right)\right). \end{array}$$

### 2.5. Infected Exposure to Virus for Susceptible Person

In studies of respiratory virus transmission, evaluating the exposure of a susceptible person to infectious aerosol particles is essential for assessing infection risk. This section quantifies the total exposure of a susceptible individual by integrating the aerosol concentration over space and time

(38) $$\begin{array}{c} Q_{\mathrm{Rx}}\left(x,y,z,t\right)=\int_{0}^{t}\int \int C_{\mathrm{Type}}\;dx\;dy\;dz\;d\tau , \end{array}$$

where $C_{\mathrm{Type}}=\left\{C_{\mathrm{speak}},C_{\mathrm{sneeze}},C_{\mathrm{cough}},C_{\mathrm{breath}}\right\}$ represents the aerosol concentration for different types of respiratory events. The equivalent receiving volume for the susceptible person’s breathing zone is modeled as a sphere, with volume $V=\frac{4}{3}\pi R_{\mathrm{Rx}}^{3}$, where $R_{\mathrm{Rx}}$ represents the radius of the sphere corresponding to the susceptible person’s face. Although a closed-form expression for pathogen exposure can, in principle, be derived through integration, the derivation is highly complex; therefore, numerical methods and machine-learning techniques are employed to evaluate the equation.

## 3. Results

In this section, numerical experiments of the model were conducted using MATLAB R2021a, with the parameter settings outlined in Table 3. The focus is on analyzing the differences between breathing modes and how related parameters influence the overall concentration within the space and the virus exposure, $Q_{\mathrm{Rx}}$, of susceptible individuals, while also accounting for the impact of the ventilation system based on the model.

### 3.1. The Effect of Droplet Size, Humidity, and Respiratory Activities on Airborne Concentration

This section analyzes the effects of particle size and RH on droplet distribution, as well as the spatial concentration under different breathing modes. The influence of variations in each parameter on the concentration is explored in detail.

**Figure 6:**
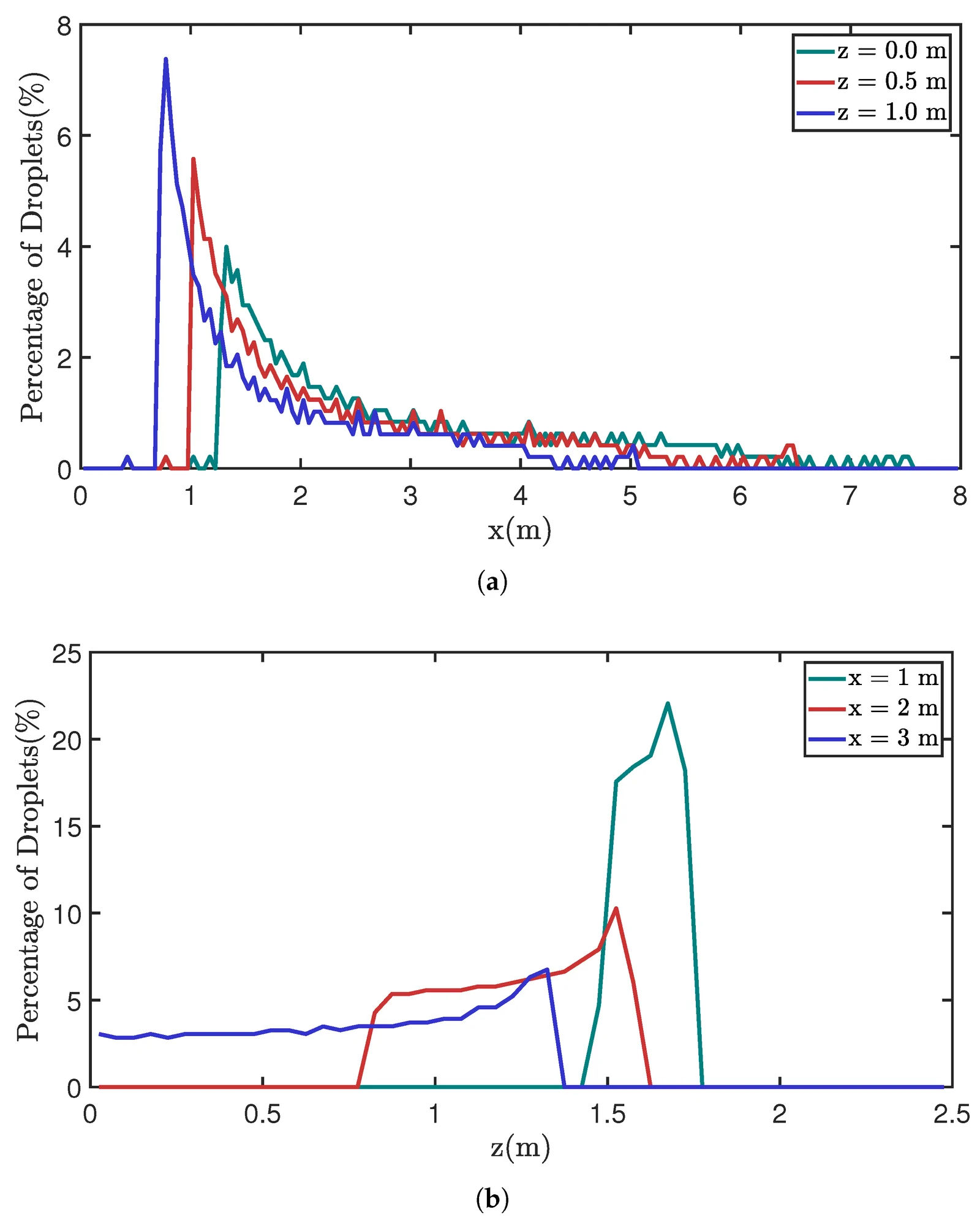
Droplets with radii ranging from 0.5 µm to 500 µm are released at a speed of 20 m/s at the coordinates (0, 1.8). (**a**) Horizontal distribution of aerosol quantity at heights *z* = 0, 0.5, and 1 m. (**b**) Distribution of aerosol quantity at different heights when *x* = 1, 2, and 3 m.

First, to investigate the effect of particle size, droplets with radii ranging from 0.5 to 500 µm are considered, with a release point of (0, 1.8) and an initial velocity of 20 m/s. The distribution characteristics of this droplet group upon landing and in the vertical plane are shown in Figure 6. In Figure 6a, the horizontal distribution reveals that, for each curve, the number of droplets is zero near the release point, with a primary deposition peak around 1 m. The number of droplets then gradually decreases as the distance increases. This deposition peak is primarily attributable to medium-sized droplets (50–200 µm), whose changing size during evaporation affects the balance between aerodynamic drag and gravitational settling. Small droplets (0.5–50 µm) dominate long-range deposition due to their prolonged suspension time, while larger droplets (200–500 µm) contribute significantly to mid-range deposition by overcoming air resistance through their larger size. Among the curves, a lower z-coordinate corresponds to a longer settling time, which allows more time for airborne transport and diffusion and consequently produces a more uniform droplet distribution. This results in a gradual decrease in peak values, with peak positions shifting backward. Figure 6b shows the vertical distribution characteristics of droplets at different horizontal positions. Each curve exhibits an upward trend in droplet distribution. The closer the curves are to the release point (e.g., x=1 m), the more concentrated the droplet distribution is in the vertical direction, with higher peaks and a distribution range closer to the height of the infected source at 1.8 m. Conversely, as the distance from the release point increases (e.g., x=3 m), the droplets have more time to diffuse during transport. Although the curve peak values decrease to around 5%, the distribution becomes more widespread, with droplets present at heights ranging from 0 to 1.4 m. Therefore, for individuals taller than 1.4 m, even when facing the infected source from which someone coughs or sneezes, maintaining a distance of about 3 m can effectively prevent exposure to the majority of droplets.

**Figure 7:**
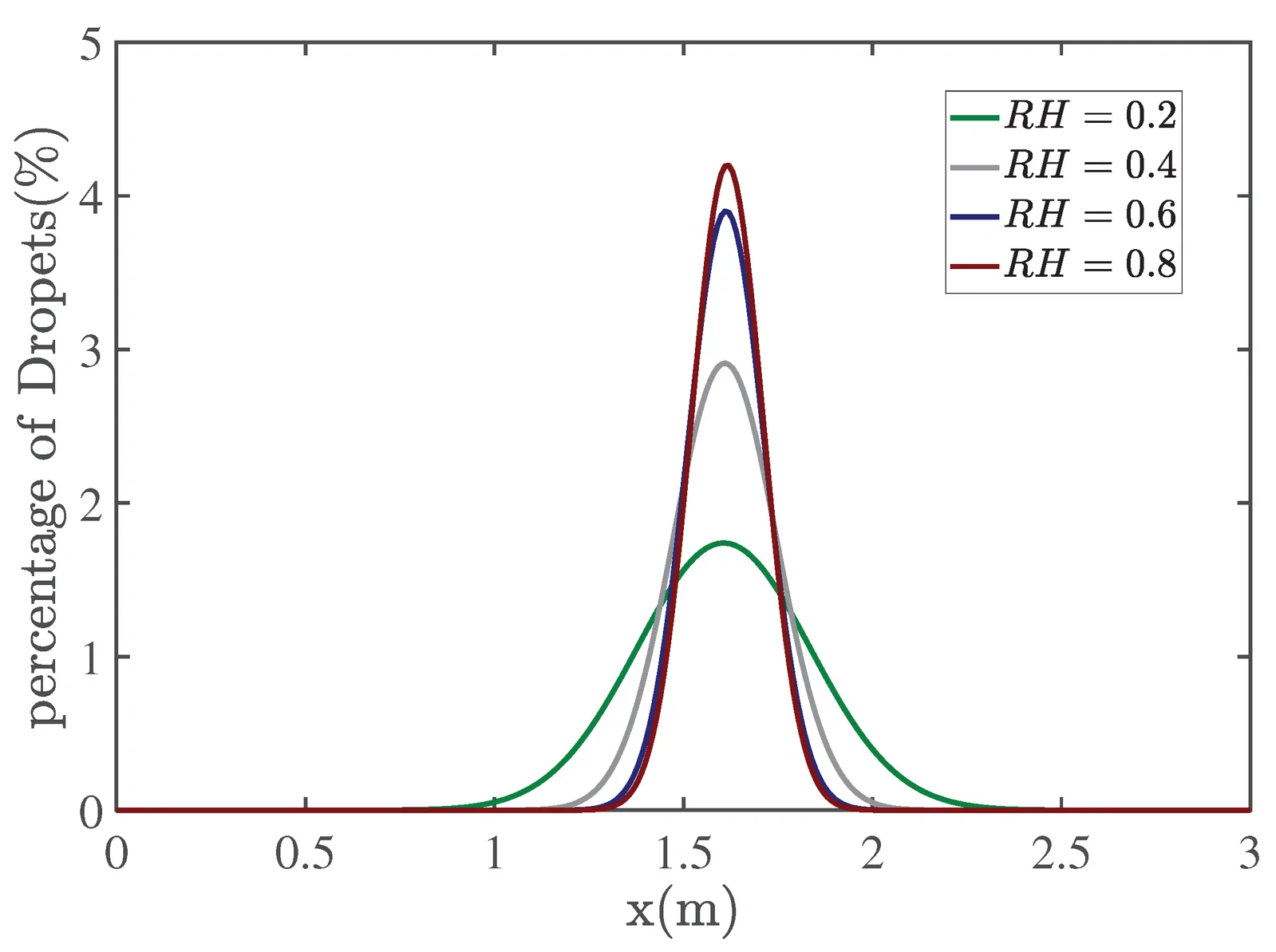
Distribution ratio of droplets expelled from an infected source at (0, 1.8) when coughing at a velocity of 20 m/s under different RH conditions.

In addition to droplet size, RH plays a pivotal role in the evaporation process, significantly influencing droplet distribution, as illustrated in Figure 7. It is evident that the peak values and steepness of the curves correlate with RH. In high-humidity environments, the difference in water-vapor pressure between the droplet surface and the ambient air is small, resulting in slower evaporation and a shorter settling time. This results in a more concentrated droplet distribution area and a steeper curve, with a peak value of approximately 4.2%. In contrast, in low-humidity environments, the evaporation rate increases, extending the suspension time of droplets. Upon landing, the peak value decreases by 2.5% compared to high-humidity conditions, but the distribution becomes more dispersed, thereby expanding the infection range. Consequently, during pandemic control, implementing necessary humidification measures in indoor environments can slow down droplet evaporation and reduce the transmission range.

**Figure 8:**
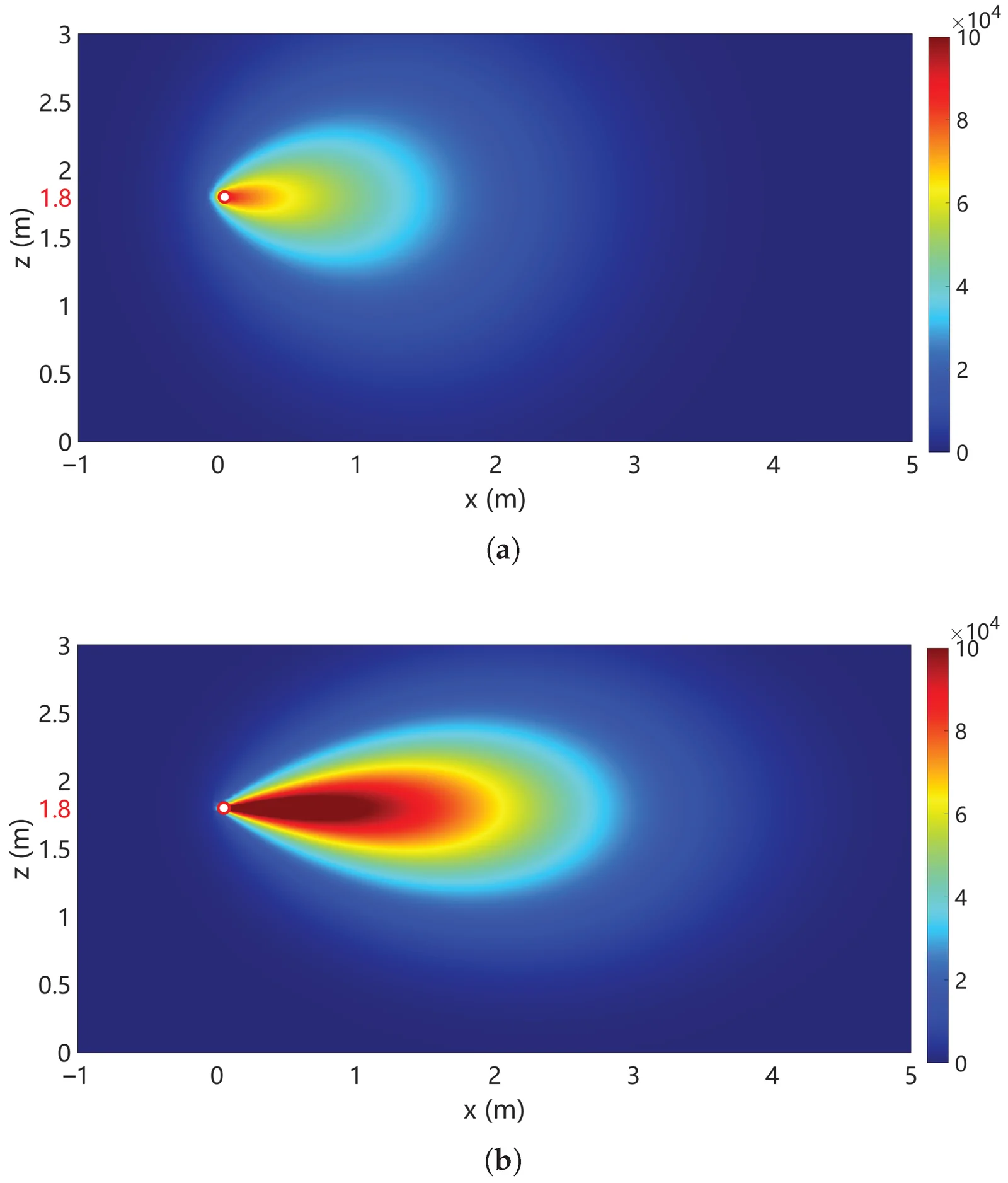
Concentration heat maps of droplets emitted during (**a**) breathing and (**b**) speaking from the infected source at (0, 1.8). The modal radii (0.8 μm and 1.6 μm) are below the gravitational threshold, so no downward trajectory occurs.

**Figure 9:**
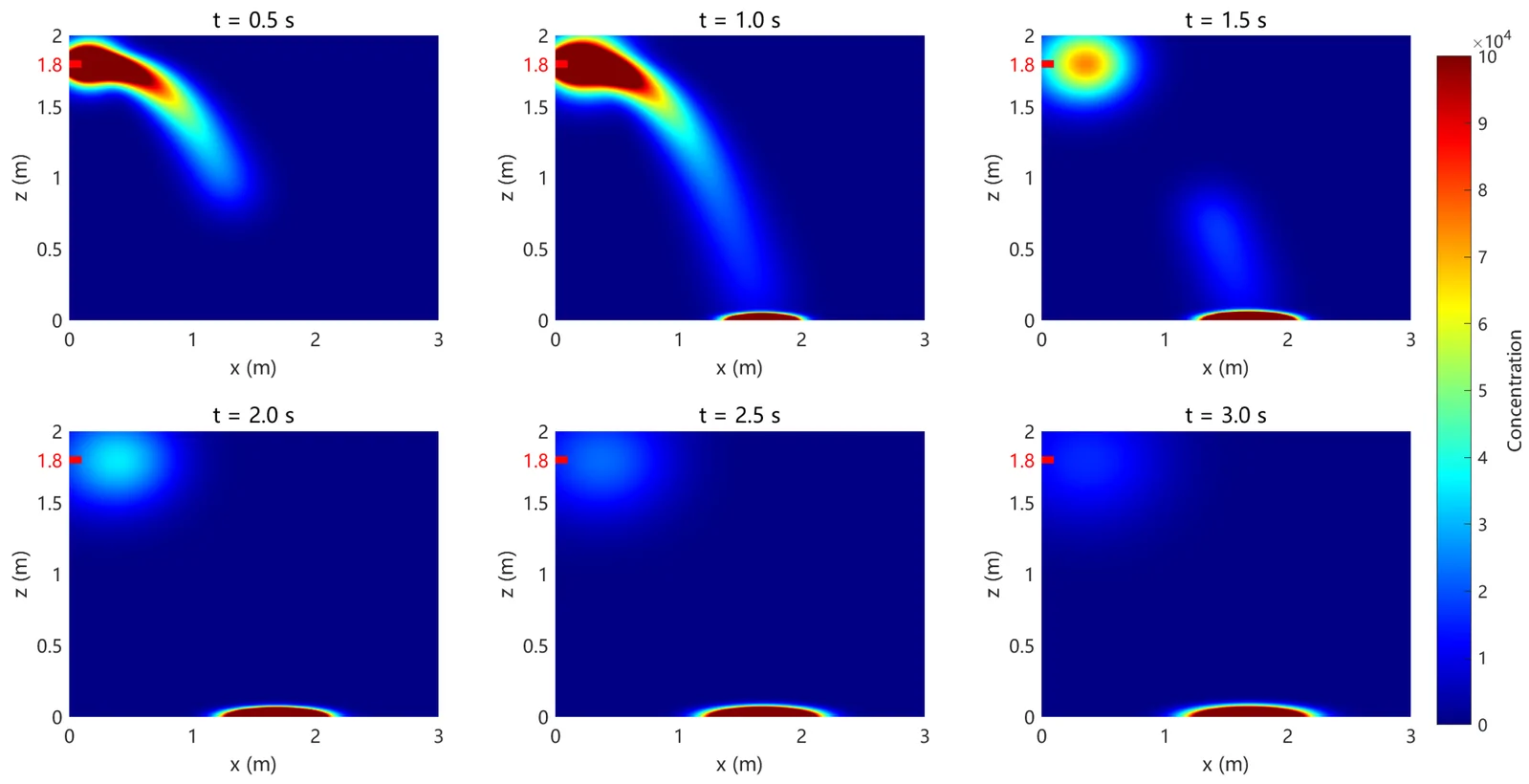
Concentration heat maps of droplets emitted during coughing from the infected source at (0, 1.8) include two particle sizes, $R_{0}=1.5$ µm and $R_{0}=74$ µm.

**Figure 10:**
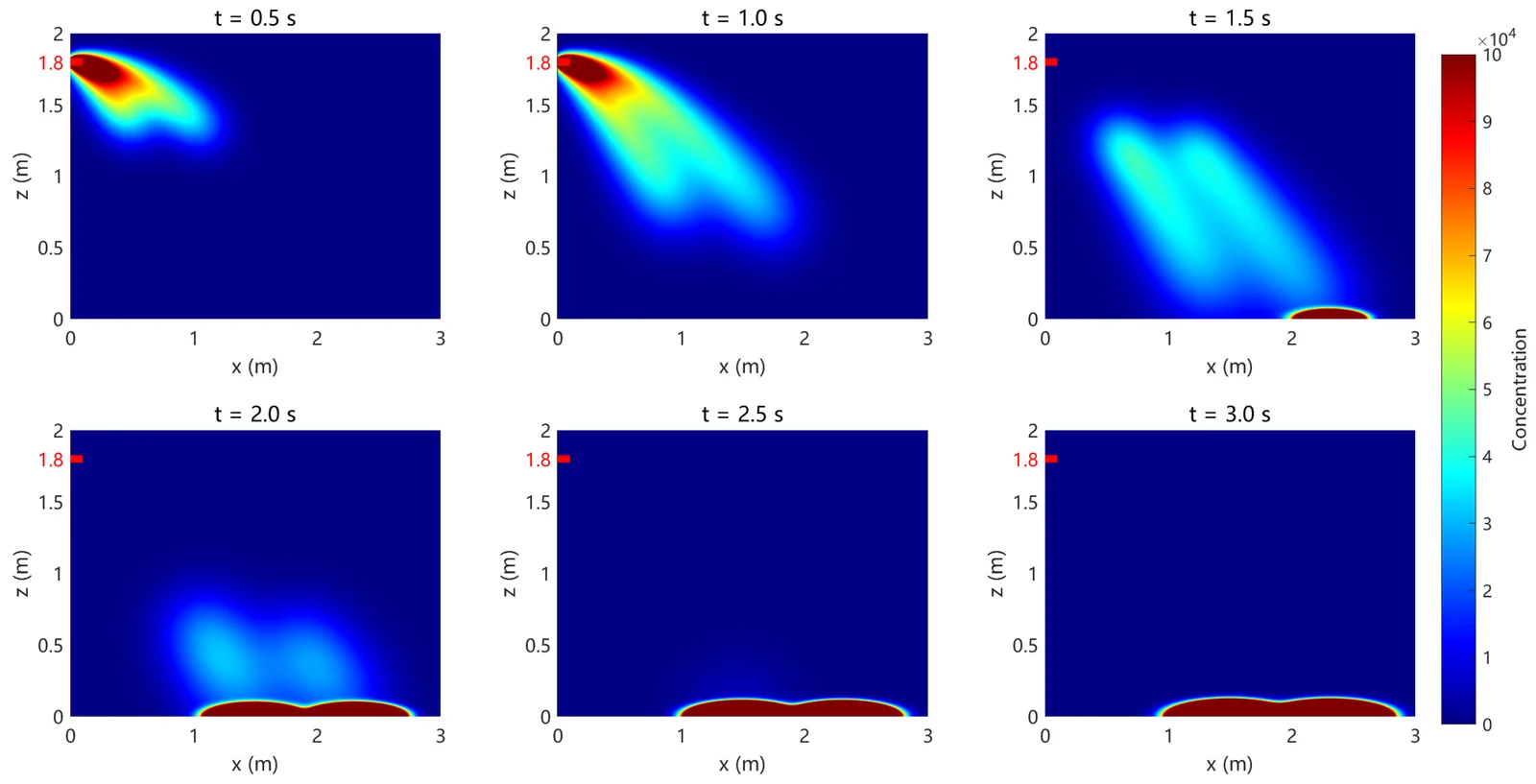
Concentration heat maps of droplets emitted during a sneeze from the infected source at (0, 1.8) include two particle sizes, $R_{0}=74.4\;$µm and $R_{0}=360.1\;$µm.

For different respiratory activities, corresponding concentration heat maps were generated to clearly illustrate the differences, as shown in Figure 8, Figure 9 and Figure 10. Figure 8 presents the concentration heat map of droplets emitted during breathing and speaking, with the infected source located at (0, 1.8). As both activities are modeled as steady-state emission processes, the droplets disperse forward in an approximately elliptical pattern, with their concentration decreasing as the distance from the infected source increases. In the case of breathing, the high-concentration area is centered around 0.5 m, with a relatively limited diffusion range. However, during speaking, a more elongated and high-concentration plume is formed, extending beyond 1 m. The distribution range of the maximum concentration is significantly larger compared to the breathing scenario, nearly double the range observed during breathing. This increase is not only due to the higher viral load expelled during speaking but also due to the more turbulent airflow, resulting in larger droplet sizes and consequently higher concentrations in the forward area. In crowded environments, particularly those containing infected sources, it is advisable to minimize prolonged communication and wear masks to slow the transmission speed and ultimately control the spread of droplets.

Figure 9 illustrates the concentration heat maps of droplets emitted during coughing, with the infected source located at (0, 1.8), at different time intervals (*t* = 0.5, 1.0, 1.5, 2.0, 2.5, 3.0 s). The droplet group consists primarily of two particle sizes: $R_{0}$ = 1.5 µm and $R_{0}$ = 74 µm, depicting their dynamic motion and diffusion in the air.

At the initial stage, high-concentration droplets are released from the infected source’s mouth. Over time, the droplet plume structure gradually becomes more sparse and diffuse, with larger droplets settling to the ground due to gravity and diffusion, causing minimal spreading upon landing. Smaller droplets remain suspended in the air; although they move at a slower speed compared to larger droplets, they generally continue to drift in the positive x direction.

Regardless of size, the concentration of droplets decreases over time due to free diffusion. Compared to breathing and speaking, coughing leads to the rapid settling of droplets, with transmission distances ranging from 1 to 2.5 m, and a significant quantity of viral droplets remaining in the landing area.

Figure 10 presents the spatial concentration distribution heat maps of droplets emitted during sneezing, with the infected source located at (0, 1.8) at various time points (*t* = 0.5, 1.0, 1.5, 2.0, 2.5, 3.0 s). The results cover two droplet sizes, $R_{0}$ = 74.4 µm and $R_{0}$ = 360.1 µm. The results show that the droplet population separates into two plumes that gradually settle, ultimately forming high-concentration deposition zones on the ground at distances of 1–3 m from the source. To prevent further transmission to susceptible individuals, it is crucial to address these high-concentration areas on the ground through ventilation measures following coughing and sneezing.

### 3.2. Virus Exposure of Susceptible Person Under Different Respiratory Activities

**Figure 11:**
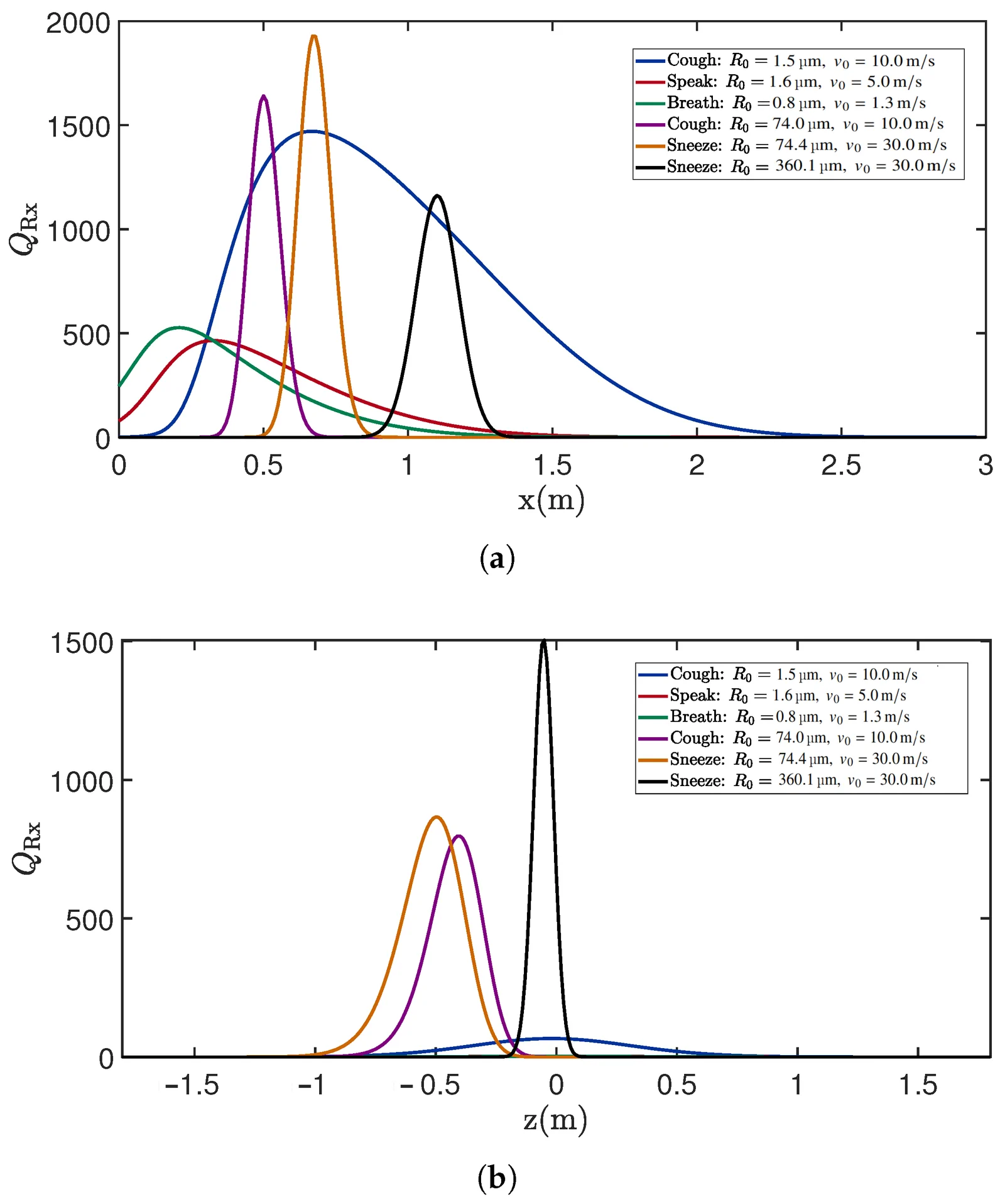
Droplets expelled from an infected source at (0, 1.8) using different breathing methods. (**a**) The relationship curve between the cumulative viral exposure of a susceptible person and *x* at z=1.8 m over 10 s. $Q_{\mathrm{Rx}}$ is obtained by spatial integration over a finite receiver sphere ($R_{\mathrm{Rx}}=0.1$ m), so the peak occurs at a near-source distance rather than at x=0. (**b**) The relationship curve between the cumulative viral exposure of a susceptible person and *z* at x=1 m over 10 s.

Two sets of data regarding the virus exposure of susceptible individuals were obtained, reflecting both their height and position, as shown in Figure 11. In the x direction, the effects of breathing and speaking are more pronounced at close range, peaking at approximately 500 at a distance of 0.25 m. The transmission distance and concentration are also influenced by the exposure time *t*; as the exposure duration increases, the peak shifts farther from the source, and the affected range expands. Among all the curves, the cumulative number of 74.4 µm droplets during sneezing is the highest, approaching 2000 particles, indicating that sneezing results in the most significant exposure of all scenarios studied. In contrast, during coughing, droplets with a radius of 1.5 µm produce the broadest transmission range, with susceptible individuals within a 0 to 2.5 m radius at risk of infection. This can be attributed to their higher initial velocity and the relatively low effect of gravity on smaller droplets, allowing them to remain suspended in the air for extended periods.

In the z direction, at a distance of 1 m from the infected source, large droplets from sneezing and coughing can still result in infection counts ranging from 1000 to 1500. Meanwhile, the curves for speaking and breathing remain nearly horizontal, indicating that droplets expelled during these activities do not reach susceptible individuals over the short exposure period considered. In contrast, smaller droplets from coughing can reach susceptible persons 1 m away due to their velocity.

### 3.3. The Effect of Ventilation on Viral Exposure of Susceptible Person

Heating, ventilation, and air-conditioning (HVAC) systems are the primary means of providing mechanical ventilation in buildings, and their effectiveness is closely related to the characteristics of the local airflow field. For instance, thermophoresis and slip flow significantly impact the transmission pathways of particles in the air. Previous studies indicate that indoor droplet transmission can be divided into two phases: an initial phase dominated by the exhaled airflow, corresponding to short-range transmission, and a subsequent phase dominated by the ambient airflow, corresponding to long-range transmission [56].

Indoor airflow dynamics mainly arise from forced ventilation systems [46]. Although various models exist for analyzing airflow, this section focuses on basic ventilation strategies and their impact on droplet concentration distribution. MV, a common indoor ventilation method, introduces fresh air at high speed from the ceiling, ensuring thorough mixing within the room and improving overall air quality [57,58]. The effect of ventilation on droplet transport is modeled by superposing a constant ventilation velocity onto the droplet velocity: $v_{x}^{\mathrm{MV}}\left(t\right)=v_{\mathrm{MV}}+v_{x}\left(t\right)$ for mixed ventilation, $v_{z}^{\mathrm{DV}}\left(t\right)=v_{\mathrm{DV}}+v_{z}\left(t\right)$ for displacement ventilation, and $v_{z}^{\mathrm{DWV}}\left(t\right)=v_{\mathrm{DWV}}+v_{z}\left(t\right)$ for downward ventilation. The superposition formulation represents the mean advective effect of the ventilation flow on the droplet cloud. The turbulent fluctuations of the indoor airflow are not resolved individually; their dispersive effect on the concentration field is instead parameterized through the turbulent diffusion coefficient $D_{\mathrm{tu}}$ (Section 2.4). This gradient-diffusion treatment is fundamentally different from the Lagrangian entrainment of droplets by resolved turbulent eddies, which requires CFD, LES, or DNS of the room airflow; a detailed discussion of the resulting applicability limits is provided in Section 5.

DV is suitable for environments with high ceilings that require cooling. The system introduces cool, clean air from the bottom of the room, enveloping heat sources and creating thermal stratification that is then exhausted from the top. The upward airflow in DV is represented by $v_{\mathrm{DV}}>0$ in the velocity superposition model.

DWV introduces cold air from the top of the room, generating an overall downward airflow that carries aerosols away from the breathing zone and exhausts them from floor-level outlets. The downward airflow is represented by $v_{\mathrm{DWV}}<0$ in the velocity superposition model.

**Figure 12:**
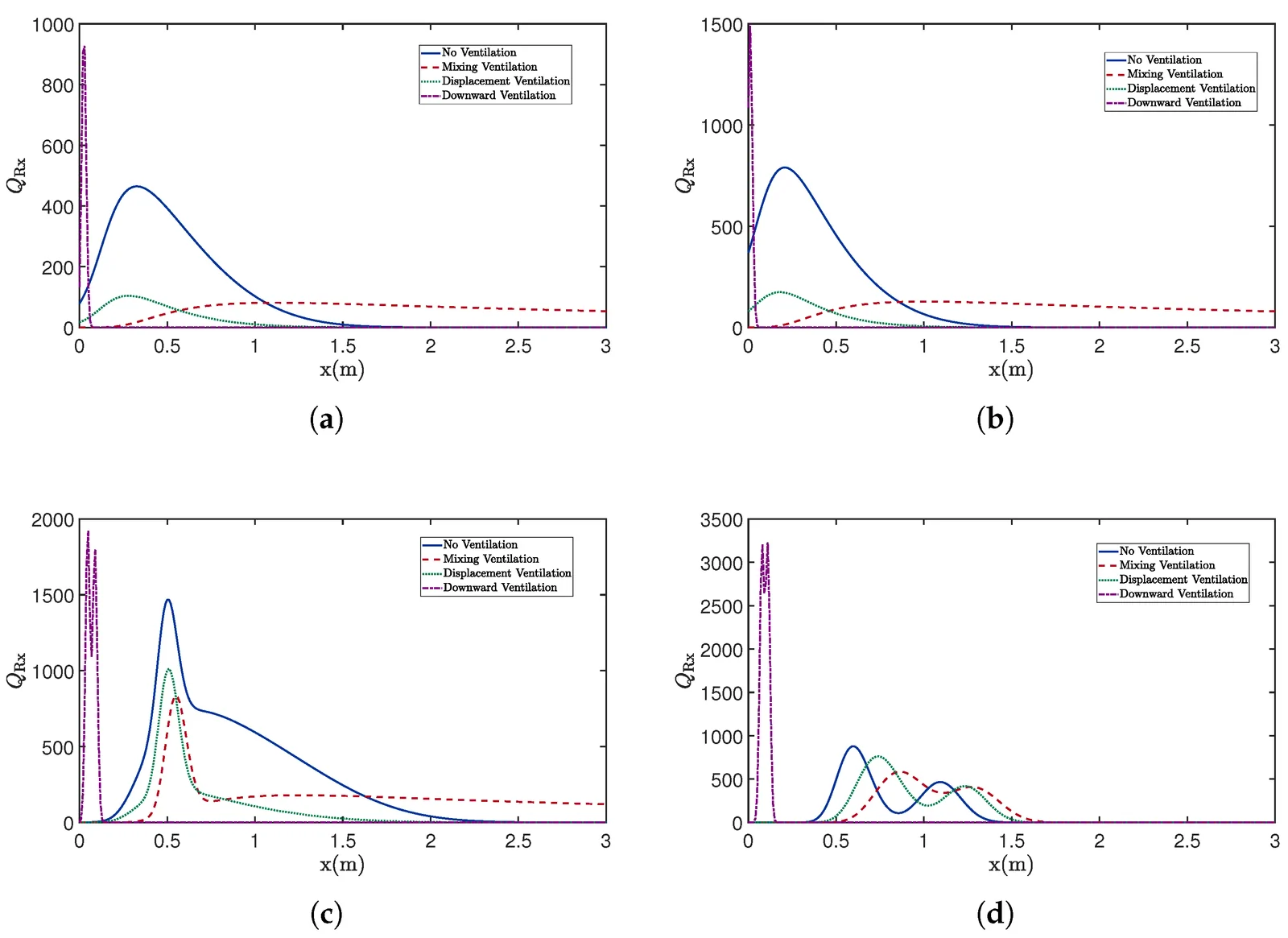
Cumulative virus exposure of susceptible individuals over 10 s as a function of position *x* under different ventilation strategies ($v_{\mathrm{MV}}=1\;m/s$, $v_{\mathrm{DV}}=1\;m/s$, $v_{\mathrm{DWV}}=-1\;m/s$): (**a**) breathing, (**b**) speaking, (**c**) coughing, (**d**) sneezing.

The impact of different ventilation strategies on viral aerosol distribution under various breathing patterns was evaluated. For each breathing mode, three typical ventilation methods (MV, DV, and DWV) were compared, along with a no-ventilation control group as the baseline. The performance of each ventilation strategy was quantitatively evaluated based on the viral exposure of susceptible individuals at different positions *x* (Figure 12).

The results indicate that MV provides a favorable overall dilution effect by promoting uniform mixing of indoor air. The ventilation comparison results shown in Figure 12 are obtained from numerical evaluation of the cumulative exposure integral $Q_{\mathrm{Rx}}$ (Equation (33)) with the ventilation-modified velocity expressions derived in this section. They are not literature data; the corresponding experimental validation using the molecular communication testbed is presented in Section 4. However, when the fresh air replacement rate is insufficient, this method may allow viral droplets to persist in distant areas, leading to a long tail phenomenon in the exposure curve. In contrast, DV, leveraging thermal stratification, creates a clean air layer in the breathing zone, significantly enhancing local ventilation efficiency. Its exposure curve shows a rapid decay, but its overall dilution capacity is more limited [59]. Finally, DWV effectively sediments droplets through directed airflow, demonstrating optimal aerosol concentration control within the breathing zone. As shown in Figure 12, under this mode, viral droplets are primarily confined to the near-field region (with small *x* values), significantly limiting long-distance transmission. This mode is particularly recommended for infection control in environments with high cleanliness requirements [14].

## 4. Testbed

To validate the theoretical model presented in Section 2, this study aims to establish a testbed for horizontal molecular communication within an indoor environment. While concentration-based transport equations are well established in fluid dynamics, the testbed experiments serve two purposes that distinguish this work from purely computational studies. First, the controlled experiments provide quantitative validation of the theoretical model under well-characterized conditions (known humidity, ventilation velocity, and emission parameters), enabling the assessment of the model’s predictive accuracy through metrics such as $R^{2}$, SSE, and RMSE. Second, the testbed demonstrates the feasibility of real-time biosensing for droplet concentration monitoring, bridging the gap between analytical modeling and practical monitoring applications. It should also be stated clearly what the testbed validation does and does not demonstrate: the strong quantitative agreement between the fitted model and the measured concentration profiles confirms that the model captures the dominant advective–diffusive mechanisms in a controlled setting, but it does not, by itself, guarantee quantitative transferability to a specific real room with non-uniform, turbulent airflow. Such transfer requires either coupling the validated model with a CFD-computed airflow field or calibrating the effective dispersion coefficient $D_{\mathrm{tu}}$ against in situ tracer-gas measurements, as discussed in Section 5. Although the current experiments use alcohol vapor as a surrogate for respiratory droplets, the platform can be extended to aerosolized saline solutions or fluorescent particle tracking in future work to more closely replicate actual droplet transport physics. Experimental trials will be conducted to verify the accuracy of the model.

### 4.1. Platform Composition

#### 4.1.1. Basic Components of the Platform

**Figure 13:**
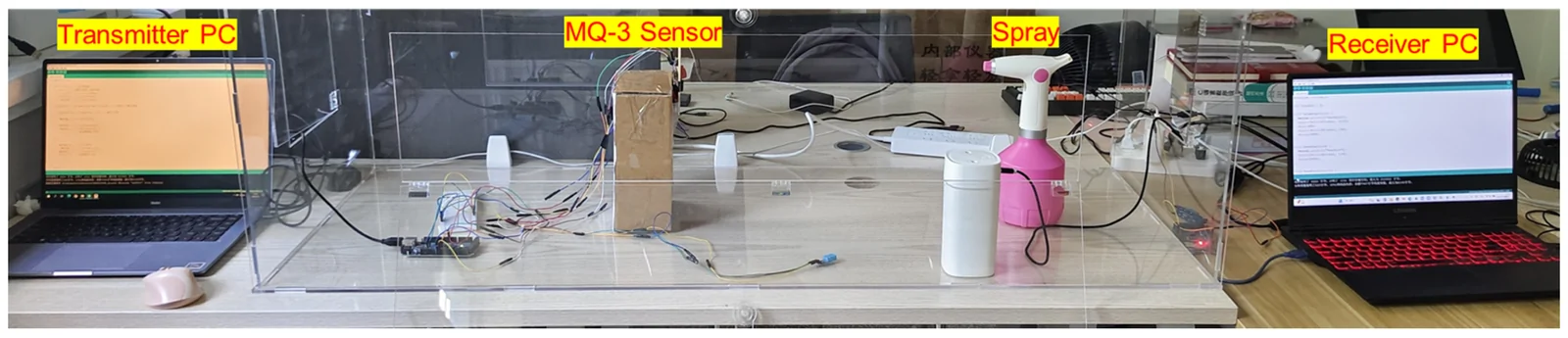
Molecular Communication Testing Platform.

**Figure 14:**
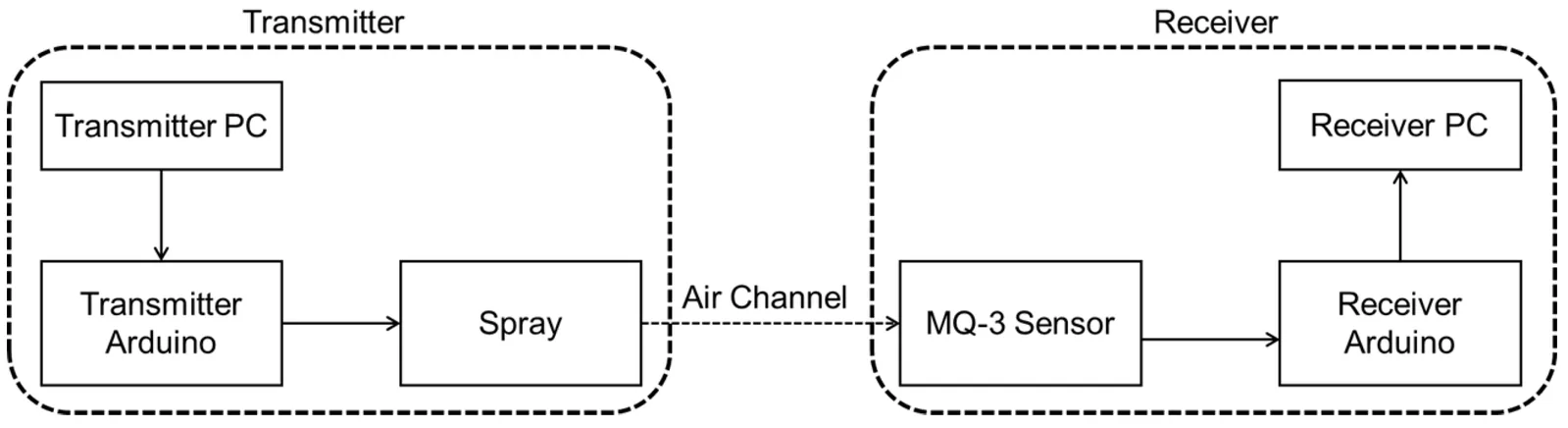
Structure of the Molecular Communication Testing Platform.

The developed horizontal molecular communication testbed takes into account factors such as breathing mode, ventilation method, and humidity. The testbed enclosure measures 1.5 m (length) × 0.5 m (width) × 0.5 m (height), constructed from high-transparency acrylic panels with operable access ports on all sides. The electric sprayer (XBR-01, Xiebingran, Yiwu, Zhejiang, China) is fixed at one end of the enclosure at a height of 0.4 m, approximating the mouth height of a seated individual in this scaled configuration. The MQ-3 alcohol sensor module (MQ-3, Hanwei Electronics Co., Ltd., Zhengzhou, China) is mounted on a movable platform along the enclosure centerline, enabling sampling at distances from 0 to 1.3 m from the transmitter. The airflow generation module consists of DC fans (12038, Gdstime Electronic Co., Ltd., Shenzhen, China) arranged in configurable positions for different ventilation modes as detailed in Section 4.1.3. Figure 13 shows a photograph of the assembled platform and Figure 14 provides a structural schematic. While the reduced chamber dimensions differ from full-scale rooms, the controlled environment allows precise characterization of individual physical mechanisms (evaporation, advection, diffusion) without the confounding influence of large-scale turbulent structures, making the testbed suitable for mechanistic model validation rather than direct room-scale prediction. The entire platform is housed in a high-transparency acrylic enclosure, with operable windows on all sides that facilitate interaction with external airflow, ensuring both experimental containment and controlled internal air currents. The platform primarily consists of a transmitter, a receiver, and an air channel, as illustrated in Figure 14.

The transmitting unit comprises a the transmitting unit comprises a transmitting PC (RedmiBook 14, 2020, Xiaomi Corporation, Beijing, China), an electric sprayer, and a microcontroller (Arduino Mega 2560, Keyes Studio, Shenzhen, China). Upon receiving instructions from the PC via the microcontroller, the system converts the information into a binary stream, controlling the sprayer to release alcohol molecules, thereby simulating the human respiratory process of aerosol droplet emission. Ethanol (alcohol) solution is selected as the tracer substance following established molecular communication practice [5], where alcohol–water mixtures serve as controllable proxies for volatile droplet dispersion. Although the density of ethanol (0.789 g/mL) differs from that of saliva (≈1.012 g/mL), the normalized concentration decay trends—which are the focus of the model validation—are governed primarily by the advection–diffusion process rather than the absolute density of the tracer; the fitting parameters $A_{1}$–$A_{5}$ absorb the resulting systematic offset. It should be noted that ethanol vapor in still air disperses by molecular diffusion, with a diffusivity on the order of $10^{-5}$ m^2^/s, whereas respiratory droplets are transported by turbulent diffusion and gravitational settling; in the fan-driven enclosure, however, spreading is dominated by the mean airflow together with the effective dispersion captured by the fitted coefficient $D_{\mathrm{tu}}$. The surrogate is therefore appropriate for validating the functional form of the normalized concentration decay, rather than for reproducing absolute droplet concentrations, which is the stated purpose of the model validation. The sprayer generates a polydisperse droplet population whose size distribution, although not individually characterized, is represented in the model through the effective emission number *N* and diffusion coefficient $D_{\mathrm{tu}}$. Parameters for signal modulation, alcohol solution concentration, release speed, and cycle duration can all be flexibly adjusted to accommodate a variety of experimental scenarios.

The air channel is formed by the internal space of the sealed acrylic enclosure and an airflow generation module consisting of multiple small DC fans. By varying the layout and power of the fans, the platform can switch between prevailing indoor ventilation strategies, including mixed ventilation, displacement ventilation, and downward air supply.

The receiving unit consists of an MQ-3 alcohol sensor, a receiving PC (Legion Y7000P, 2020, Lenovo Group Limited, Beijing, China), and a microcontroller. The MQ-3 alcohol sensor first generates a digital reading ranging from 0 to 1023 in response to the alcohol vapor concentration and then transmits the reading to the receiving microcontroller. Finally, the microcontroller demodulates the information from the alcohol sensor into a binary output of “0” or “1”, which is then displayed on the screen of the receiving PC. The MQ-3 is a metal-oxide-semiconductor sensor with a nominal detection range of 0.05–10 mg/L for ethanol vapor; the sensor was calibrated against known ethanol concentrations prior to each experimental session, and its output was recorded as normalized values (0–1023) for signal demodulation. Model validation relies on normalized concentration decay trends, which are independent of the absolute concentration scale. The 1:1 and 1:2 alcohol-to-water dilution ratios for sneeze and cough simulations, respectively, produce distinct signal levels corresponding to the different emission intensities. The testbed humidity of 50% RH was maintained using a commercial humidifier controlled by a digital humidity sensor in a feedback loop; all calibrations and experiments were conducted at the same nominal RH to ensure consistent sensor baseline conditions. The ventilation airflow velocities were measured at the fan outlet using a hot-wire anemometer and are reported with the corresponding ventilation experiments. The placement of the receiver is determined based on the specific experimental scenario, allowing for molecular concentration sampling at multiple critical points within the space.

#### 4.1.2. Horizontal Communication Process and Signal Modulation

During the experiment, the text “hello” was transmitted from the transmitting PC, with each character converted into its corresponding ASCII code sequence. Subsequently, according to the frame structure predefined in the next section, a frame header “11” is automatically added to the beginning of the bit stream for each ASCII character, and a frame tail “00000” is appended at the end to ensure synchronization of the signal at the transmitter.

Considering that the alcohol concentration in the chamber does not dissipate rapidly, this platform employs differential concentration shift keying as the signal modulation technique. When transmitting a bit “1”, the sprayer operates for 100 ms, followed by a 400-ms interval to simulate a droplet release and facilitate molecular dispersion in a low-speed airflow. Conversely, when transmitting a bit “0”, the sprayer emits no molecules and waits for 500 ms.

Once the alcohol molecules are released, they propagate horizontally within the enclosure. The MQ-3 sensor at the receiving end responds to the molecular concentration and outputs an electrical signal ranging from 0 to 1023. The receiving microcontroller determines whether the current bit is “0” or “1” by analyzing the difference between the sensor readings obtained in two consecutive time slots, thereby enabling real-time signal demodulation. All restored binary information is then reconstructed into its original character format via the receiving PC, completing the end-to-end information transmission process.

In this platform, each character is encoded using an 8-bit ASCII format. A complete information frame consists of three parts: two bits of “1” as the header, 8 bits of ASCII code as the effective data, and 5 bits of “0” as the frame tail. For instance, to transmit the letter “h”, which has an ASCII code of “01101000”, the corresponding frame bit stream would be “110110100000000”, totaling 15 bits. Each bit is transmitted within a 500 ms time slot; therefore, transmitting the complete 15-bit frame requires 7.5 s, corresponding to a transmission rate of 2 bit/s.

#### 4.1.3. Ventilation Design

To simulate the effects of indoor ventilation on respiratory droplet transport, the testbed integrates a multi-position fan array with an airflow control module, enabling precise regulation of three typical ventilation modes: mixed ventilation, displacement ventilation, and downward ventilation.

**Figure 15:**
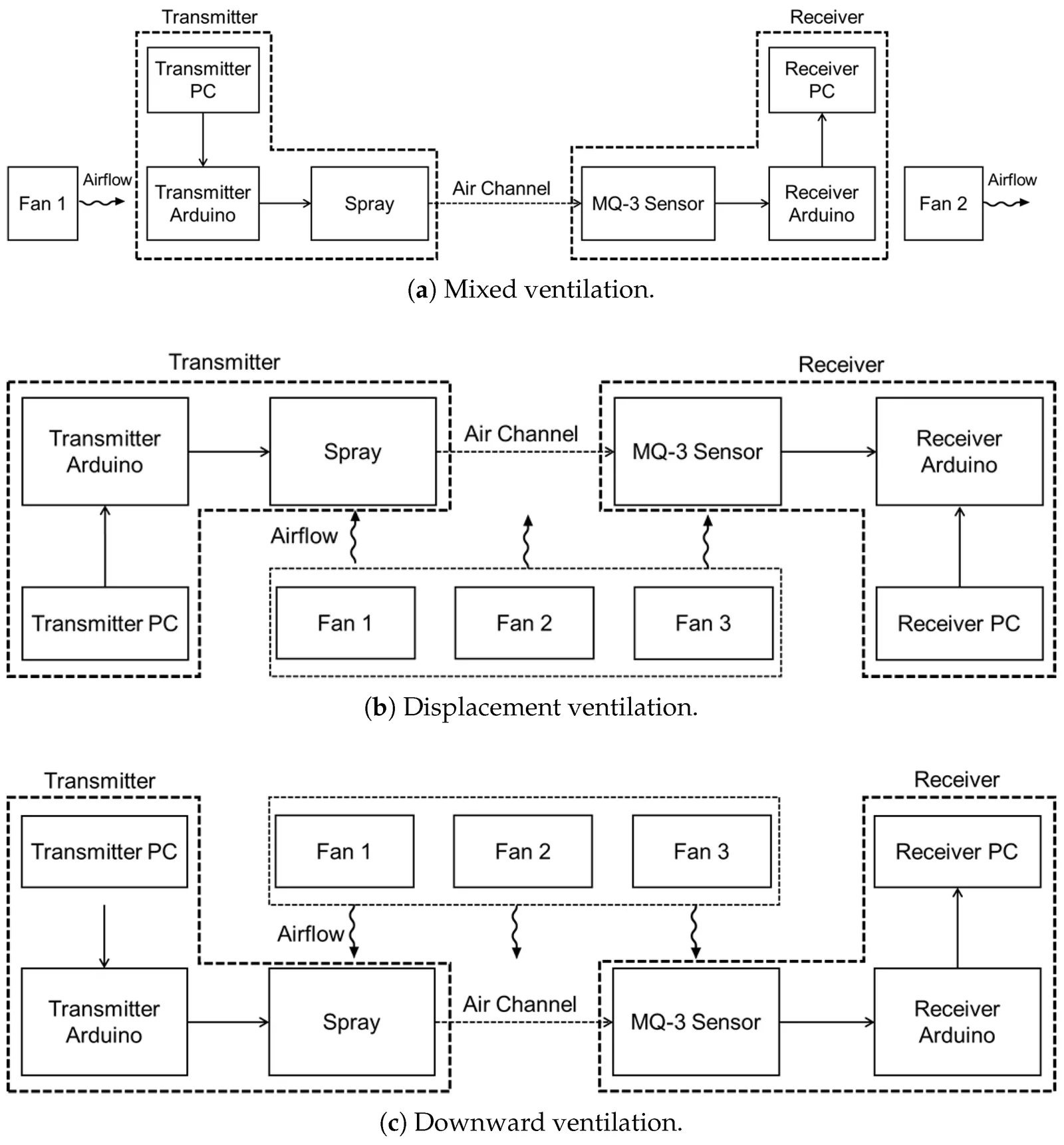
Design diagrams of the three ventilation configurations on the molecular communication testbed. The fan layouts and airflow directions for configurations (**a**–**c**) are indicated in the corresponding subfigures.

The mixed ventilation design aims to replicate conventional horizontal airflow scenarios within indoor environments, with the layout and airflow characteristics illustrated in Figure 15a. Fan groups are positioned to the left of the transmitter and to the right of the receiver. Their coordinated operation generates a horizontal, directional airflow field along the primary droplet transport pathway. During the experiment, the speed differential between the fans on each side can be adjusted to simulate various turbulence intensities of mixed ventilation environments, accommodating the ventilation characteristics of ordinary indoor spaces such as offices and conference rooms, thereby investigating the dilution effect of airflow mixing on droplets.

The displacement ventilation design is based on the principle of thermal stratification, as illustrated in Figure 15b. The platform is equipped with three sets of fans located at the bottom of the enclosure between the transmitter and receiver. Supplying air from the bottom and exhausting it from the top generates a vertically rising airflow, allowing clean air to enter at floor level, rise gradually, and be discharged through the upper outlets. This effectively simulates the floor supply displacement ventilation mode commonly found in actual scenarios. This design is applicable for environments with high air quality requirements, such as hospital wards and laboratories, and can be utilized to analyze the effects of vertically rising airflow on droplet deposition and stratified distribution.

The downward supply air design aims to expedite the rapid sedimentation of droplets through directed airflow, with its structure and physical model illustrated in Figure 15c. The platform is equipped with three sets of fans positioned at the top of the enclosure between the transmitter and receiver, generating a vertically downward ventilation. This airflow, combined with exhaust fans located on both sides at the bottom, creates a flow field characterized by top supply and bottom discharge, directly impacting horizontally propagating droplet molecules to accelerate their sedimentation. This mode is suitable for scenarios requiring rapid control of pollutant dispersion, such as in infectious disease wards and clean rooms, and can be compared with the effects of displacement ventilation for analytical purposes.

### 4.2. Theory and Model Fitting

To validate the previously established kinetic model for the horizontal transport of respiratory droplets, which accounts for droplet evaporation, gravitational settling, air resistance, and ventilation effects, this section compares the theoretical predictions with experimental data obtained using the constructed horizontal molecular communication test platform.The experimental design employs a controlled single-variable method, using nonlinear least squares to optimize the key parameters of the theoretical model. Fit quality is evaluated using metrics such as the coefficient of determination ($R^{2}$), the sum of squared errors (SSE), and the root mean square error (RMSE), thereby verifying the applicability of the theoretical model in complex indoor environments.

#### 4.2.1. Distance–Concentration Relationship Under Different Breathing Modes

Due to the instantaneous nature of the sprayer bottle’s emission, a continuous and stable emission process could not be simulated; therefore, we only performed fitting experiments for two breathing modes: coughing and sneezing. We differentiated between these two modes by altering the fan speed settings and the concentration of the alcohol solution.For the coughing scenario, the fan was set at a medium speed, and the alcohol solution was prepared with a ratio of 1:2 for alcohol molecules to water. In the sneezing scenario, the fan was set to high speed, and the alcohol solution had a 1:1 ratio of alcohol molecules to water. We subsequently conducted experiments for both scenarios.

Based on the previously established kinetic model for the horizontal propagation of droplets, we derived molecular concentration expressions for the two breathing modes while considering the effects of gravity, air resistance, and evaporation. This includes the gravitational influence and the concentration ratio of the alcohol solution, specifically for larger droplet radii

(39) $$\begin{array}{ccc} C_{\mathrm{Type}}\left(x,y,z,t\right) & = & \frac{N}{{\left(4\pi D_{\mathrm{tu}}t\right)}^{3/2}}\exp\left(-\frac{{\left(x-s_{x}\left(t\right)\right)}^{2}+y^{2}}{4D_{\mathrm{tu}}t}\right)\\ & & \times \left(\exp\left(-\frac{{\left(z-h-s_{z}\left(t\right)\right)}^{2}}{4D_{\mathrm{tu}}t}\right)+\exp\left(-\frac{{\left(z+h-s_{z}\left(t\right)\right)}^{2}}{4D_{\mathrm{tu}}t}\right)\right). \end{array}$$

Here, $C_{\mathrm{Type}}\left(x,y,z,t\right)$ denotes the droplet concentration at position (x,y,z) and time *t* under a given respiratory activity, while *N* denotes the number of droplets released from the sprayer bottle under the corresponding coughing or sneezing condition. In this context, we focus solely on the scenarios of coughing and sneezing. The variable *h* refers to the initial release height of the droplets, which is fixed at 0.4m in the experiment, corresponding to the height of the human mouth and nose. To investigate the distance–concentration relationship under different respiratory activities, the emission point was fixed at (0,0,0.4), and the receiver was positioned at different locations along the x-axis, ranging from (0,0,0.4) to (1.5,0,0.4). The resulting measurements were used to evaluate the variation in concentration with *x* at t=1 s. Given that the theoretical model assumes “uniform airflow and no boundary interference,” there may be deviations due to the limited space of the actual acrylic box of the experimental platform (such as wall adsorption and airflow boundary layer effects). To better fit the experimental scenarios, five fitting parameters, $A_{1}$–$A_{5}$, were introduced into the model. By substituting t=1s, y=0, z=0.4, the specific fitting expression takes the form

(40) $$\begin{array}{ccc} C_{\mathrm{Type}}^{\mathrm{fit}}\left(x\right) & = & \frac{N}{{\left(A_{1}\pi \right)}^{3/2}}\exp\left(-\frac{{\left(x-\int_{0}^{1}v_{x}\left(\tau \right)\;d\tau \right)}^{2}}{A_{1}}\right)\\ & & \times \left(\exp\left(-\frac{{\left(-\int_{0}^{1}v_{z}\left(\tau \right)\;d\tau \right)}^{2}}{A_{1}}\right)+\exp\left(-\frac{{\left(0.8-\int_{0}^{1}v_{z}\left(\tau \right)\;d\tau \right)}^{2}}{A_{1}}\right)\right), \end{array}$$

where $v_{x}\left(\tau \right)=\frac{1}{A_{2}\left(1-\sqrt{1-A_{3}\tau }\right)+\frac{1}{v_{0}}}$, $v_{z}\left(\tau \right)=A_{4}\tanh\left(A_{5}\tau \right)$. The parameters $A_{1}$ to $A_{5}$ correspond to $4D_{\mathrm{tu}}$, $\frac{3R_{0}\rho_{a}K}{4\rho_{d}\theta \left(1-\mathrm{RH}\right)}$, $\frac{\theta }{R_{0}^{2}}\left(1-\mathrm{RH}\right)$, $\sqrt{\frac{8g\left(\rho_{a}-\rho_{d}\right)R_{0}}{3\rho_{a}K}}$, and $\sqrt{\frac{3g\left(\rho_{a}-\rho_{d}\right)\rho_{a}K}{8\rho_{d}^{2}R_{0}}}$, respectively.

**Figure 16:**
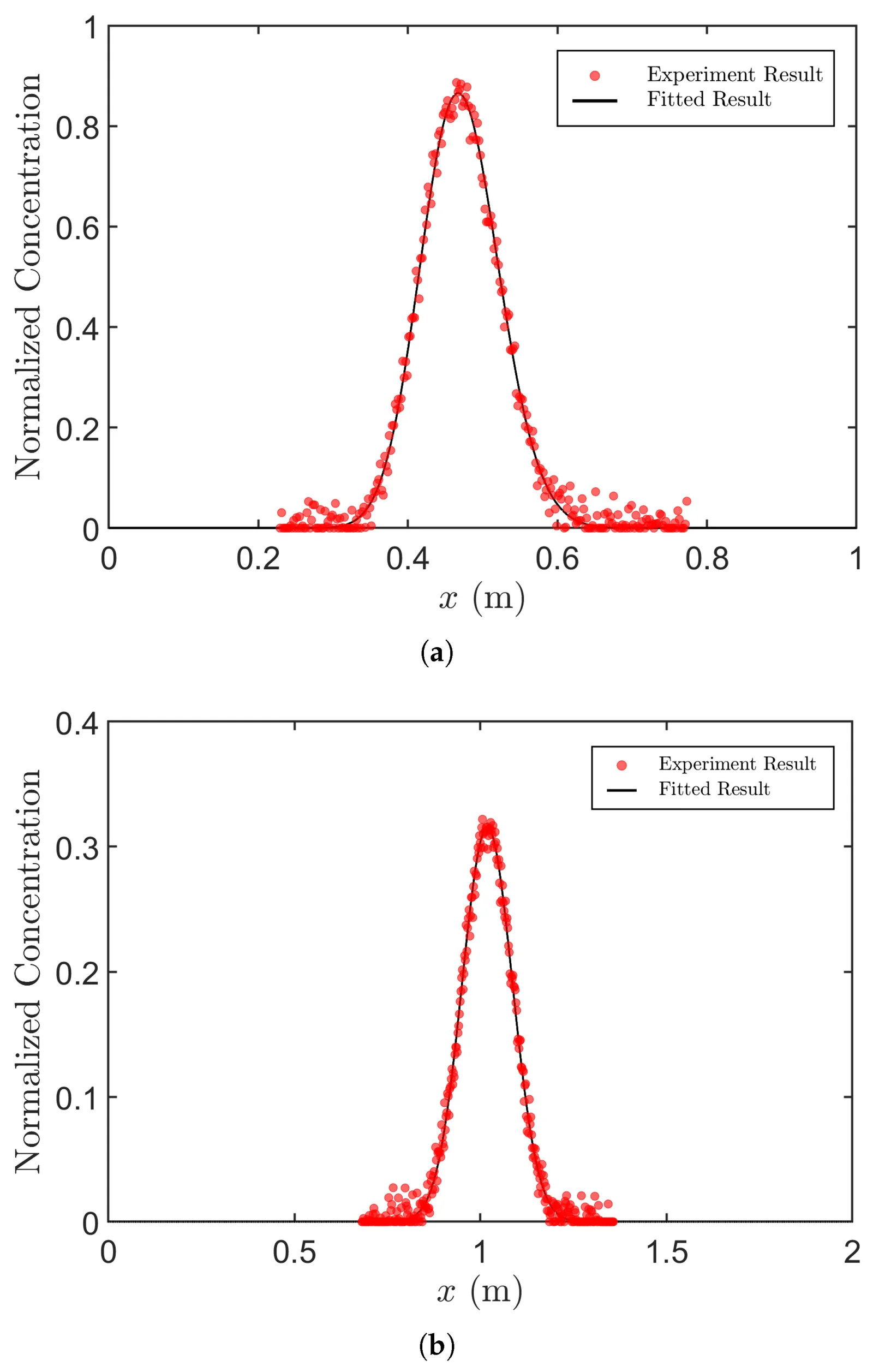
Fitting Results for Two Breathing Scenarios. (**a**) Coughing. (**b**) Sneezing in the X Direction. Data were normalized by each dataset’s maximum to compare decay trends independently of the absolute emission numbers.

Concentration data for both respiratory activities were obtained from the platform experiments. To eliminate the influence of differences in the initial number of emitted droplets when comparing the concentration distributions for coughing and sneezing, both the experimental data and the model-fitting results were normalized. Figure 16 shows the normalized experimental data and fitting curves for the coughing (a) and sneezing (b) breathing modes.

As shown in these figures, the normalized concentrations in both scenarios decrease exponentially with increasing horizontal distance. The fitted curves agree closely with the normalized experimental data, further supporting the model’s ability to characterize the horizontal transport of droplets under different respiratory activities. Table 4 summarizes the fitting parameters and model-performance metrics for the two respiratory activities. The $R^{2}$ values are close to 1, whereas the SSE and RMSE values are relatively low, quantitatively demonstrating the goodness of fit of the model.

In the case of coughing, the parameters are set as follows: $A_{1}=4.0\times 10^{-5}$, $A_{2}=2.26\times 10^{3}$, $A_{3}=4.2\times 10^{-4}$, $A_{4}=5.90$, $A_{5}=1.66$. By combining these parameters with the fixed parameters from the experiment, the fitting calculation formula can be derived as follows

(41) $$\begin{array}{ccc} C_{\mathrm{cough}}^{\mathrm{fit}}\left(x\right) & = & \frac{10^{15}}{{\left(4.0\times 10^{-5}\pi \right)}^{3/2}}\exp\left(-\frac{{\left(x-0.5\right)}^{2}}{4.0\times 10^{-5}}\right). \end{array}$$

Similarly, substituting the parameters for the sneezing scenario yields the following fitting calculation formula

(42) $$\begin{array}{ccc} C_{\mathrm{sneeze}}^{\mathrm{fit}}\left(x\right) & = & \frac{10^{16}}{{\left(3.8\times 10^{-5}\pi \right)}^{3/2}}\exp\left(-\frac{{\left(x-0.5\right)}^{2}}{3.8\times 10^{-5}}\right). \end{array}$$

#### 4.2.2. Distribution of Alcohol Molecules Under Different Humidity Conditions

To investigate the distance–concentration relationship under different humidity conditions, we focus on the coughing scenario. For the horizontal propagation of respiratory virus droplets, this study establishes a theoretical model for the temporal and spatial concentration distribution, incorporating macroscopic mass conservation, kinetic assumptions, and the physical properties of droplets, while accounting for the influences of resistance, evaporation, and gravity on molecular motion. To validate the RH variable, the concentration solution for droplets released in pulses at position (x,y,z) and time *t* is given by

(43) $$\begin{array}{ccc} C_{\mathrm{RH}}\left(x,y,z,t\right) & = & \frac{N_{\mathrm{cough}}}{{\left(4\pi D_{\mathrm{tu}}t\right)}^{3/2}}\exp\left(-\frac{{\left(x-s_{x}\left(t\right)\right)}^{2}+y^{2}}{4D_{\mathrm{tu}}t}\right)\\ & & \times \left(\exp\left(-\frac{{\left(z-h-s_{z}\left(t\right)\right)}^{2}}{4D_{\mathrm{tu}}t}\right)+\exp\left(-\frac{{\left(z+h-s_{z}\left(t\right)\right)}^{2}}{4D_{\mathrm{tu}}t}\right)\right). \end{array}$$

At the emission point, the same location as in the fitting experiments for different breathing modes is used. Regarding the variation of RH, the receiver is arranged horizontally along the x-axis from (0,0,0.4)m to (1,0,0.4)m to collect response signals at different ground positions. Humidity levels of RH=40%, 60%, and 80% are achieved using a humidifier and humidity sensor, with all other parameters consistent with those in the previous section on coughing experiments.

Similarly, we introduce five fitting parameters, $A_{1}$–$A_{5}$, to refine the model. By substituting in t=1s, y=0, z=0.4, v=20, and $N=10^{15}$, the adjusted fitting formula is as follows

(44) $$\begin{array}{ccc} C_{\mathrm{RH}}^{\mathrm{fit}}\left(x\right) & = & \frac{10^{15}}{{\left(A_{1}\pi \right)}^{3/2}}\exp\left(-\frac{{\left(x-\int_{0}^{1}v_{x}\left(\tau \right)\;d\tau \right)}^{2}}{A_{1}}\right)\\ & & \times \left(\exp\left(-\frac{{\left(-\int_{0}^{1}v_{z}\left(\tau \right)\;d\tau \right)}^{2}}{A_{1}}\right)+\exp\left(-\frac{{\left(0.8-\int_{0}^{1}v_{z}\left(\tau \right)\;d\tau \right)}^{2}}{A_{1}}\right)\right), \end{array}$$

where

$$v_{x}\left(\tau \right)=\frac{\left(1-\mathrm{RH}\right)}{A_{2}\left(1-\sqrt{1-A_{3}\left(1-\mathrm{RH}\right)\tau }\right)+0.05\left(1-\mathrm{RH}\right)},\;v_{z}\left(\tau \right)=A_{4}\tanh\left(A_{5}\tau \right).$$

The parameters $A_{1}$ to $A_{5}$ correspond to $4D_{\mathrm{tu}}$, $\frac{3R_{0}\rho_{a}K}{4\rho_{d}\theta }$, $\frac{\theta }{R_{0}^{2}}$, $\sqrt{\frac{8g\left(\rho_{a}-\rho_{d}\right)R_{0}}{3\rho_{a}K}}$, and $\sqrt{\frac{3g\left(\rho_{a}-\rho_{d}\right)\rho_{a}K}{8\rho_{d}^{2}R_{0}}}$, respectively.

**Figure 17:**
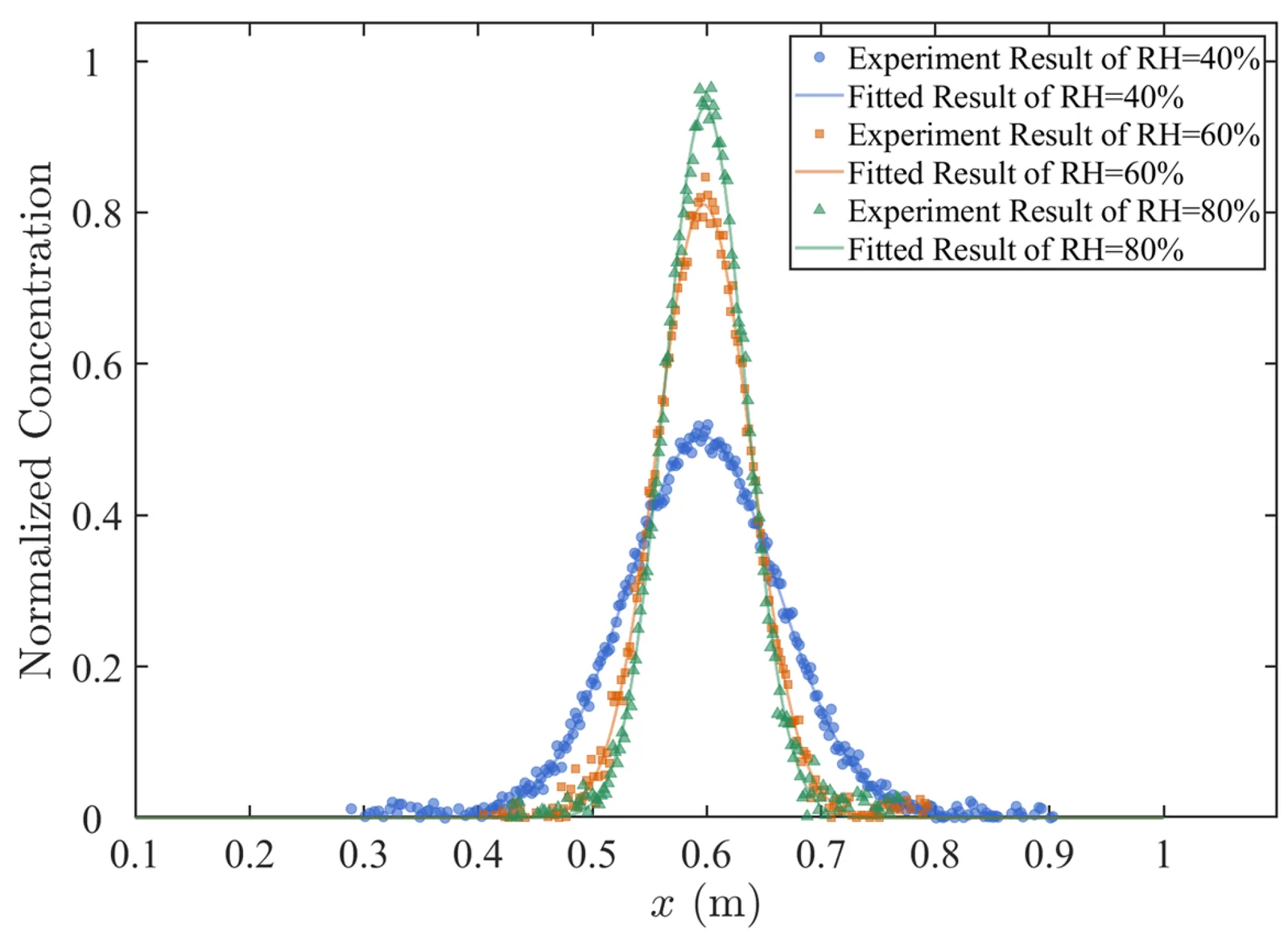
Fitting Results Under Different Humidity Conditions in Coughing Scenario.

To facilitate comparison of the horizontal concentration distributions of alcohol molecules under different humidity conditions and eliminate the influence of differences in absolute concentration, the experimental data and model-fitting results for each RH condition were normalized. Figure 17 illustrates the normalized model-fitting results and experimental data under the conditions of RH=40%, RH=60%, and RH=80%.

From the figures, it can be observed that the theoretical prediction curves, after normalization, maintain a high level of consistency with the measured experimental data. Furthermore, as RH increases, the concentration decreases more rapidly with horizontal distance, suggesting that higher humidity reduces the horizontal transport range of alcohol molecules. This further validates that the established model can accurately describe the effects of humidity variation on the distribution characteristics of alcohol molecules’ horizontal propagation. Table 5 lists the fitting parameters and model performance metrics under each humidity condition, with $R^{2}$ values all exceeding 99%. The SSE and RMSE values decrease with increasing RH, indicating closer agreement between the model predictions and experimental data under the tested high-humidity conditions.

When RH = 40%, by substituting the fitting parameters $A_{1}=4.0\times 10^{-5}$, $A_{2}=1.36\times 10^{3}$, $A_{3}=7.01\times 10^{-4}$, $A_{4}=5.90$, $A_{5}=1.66$, along with the fixed parameters from the experiment into the fitting formula, we obtain

(45) $$\begin{array}{ccc} C_{\mathrm{RH}=40\%}^{\mathrm{fit}}\left(x\right) & = & \frac{10^{15}}{{\left(4.0\times 10^{-5}\pi \right)}^{3/2}}\exp\left(-\frac{{\left(x-0.5\right)}^{2}}{4.0\times 10^{-5}}\right). \end{array}$$

Similarly, by substituting the parameters for RH = 60% and RH = 80%, we derive the corresponding concentration fitting formulas

(46) $$\begin{array}{ccc} C_{\mathrm{RH}=60\%}^{\mathrm{fit}}\left(x\right) & = & \frac{10^{15}}{{\left(3.5\times 10^{-5}\pi \right)}^{3/2}}\exp\left(-\frac{{\left(x-0.5\right)}^{2}}{3.5\times 10^{-5}}\right), \end{array}$$

(47) $$\begin{array}{ccc} C_{\mathrm{RH}=80\%}^{\mathrm{fit}}\left(x\right) & = & \frac{10^{15}}{{\left(3.1\times 10^{-5}\pi \right)}^{3/2}}\exp\left(-\frac{{\left(x-0.5\right)}^{2}}{3.1\times 10^{-5}}\right). \end{array}$$

#### 4.2.3. Distance–Concentration Relationship Under Different Ventilation Modes

To systematically investigate the effects of different ventilation strategies on respiratory droplet transport, this study considers three typical indoor ventilation modes—mixed, displacement, and downward ventilation—with the no-ventilation condition serving as the control. Ventilation control is achieved through multiple small DC fans in the molecular communication testing platform. The experiments utilize two breathing modes: coughing and sneezing. The emission point is located at the same coordinates as in the fitting experiments for different breathing modes, at (0,0,0.4)m, while the receiver is arranged along the x-axis from (0,0,0.4)m to (3,0,0.4)m, with environmental humidity set at RH=50%. The specific parameters are shown in Table 6.

**Figure 18:**
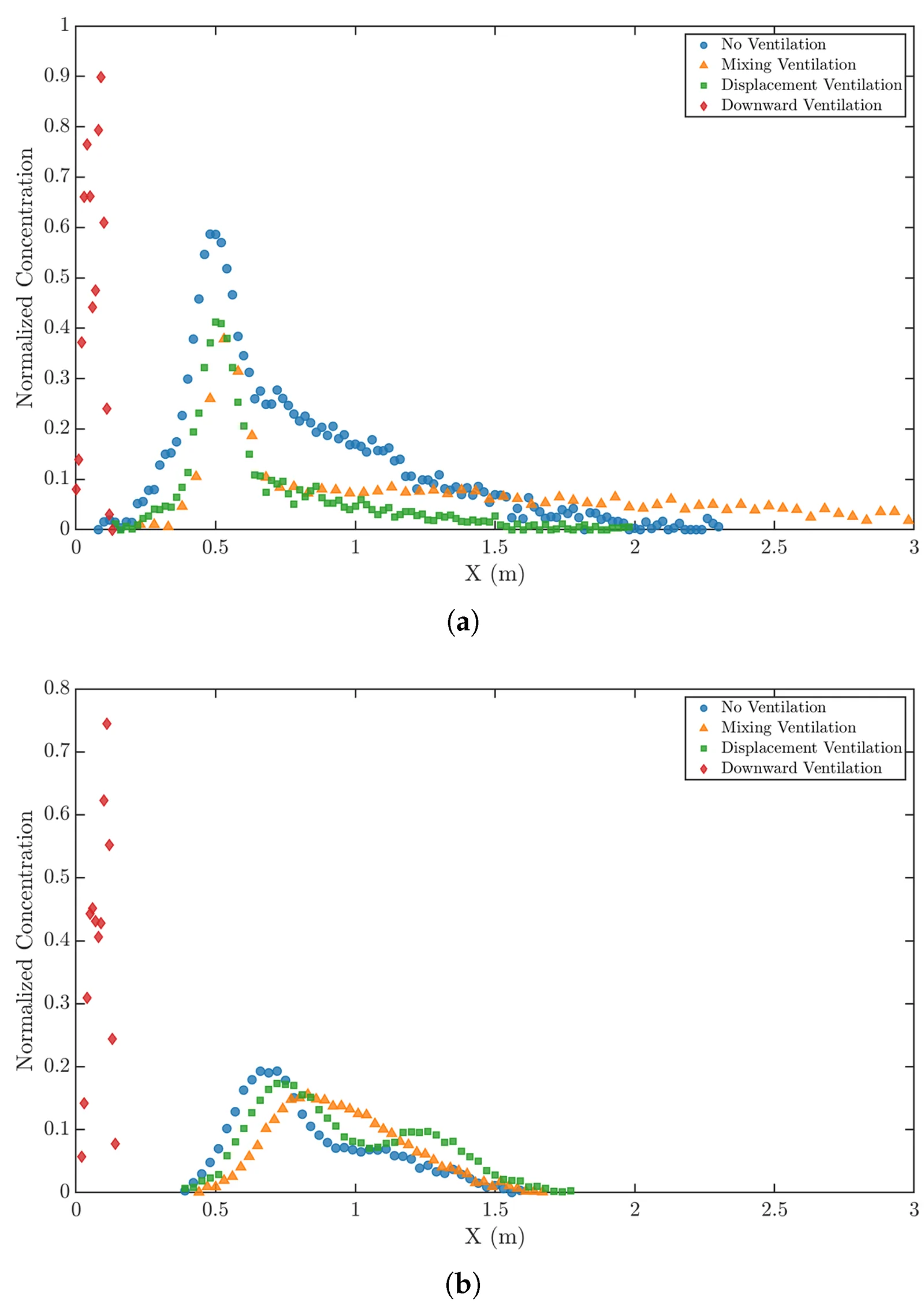
Results Under Different Ventilation Conditions for Two Breathing Scenarios. (**a**) Coughing. (**b**) Sneezing. Data collected within the 1.5 m testbed show the concentration decaying to near zero; data beyond 1.5 m represent the predicted asymptotic tail of the distribution.

The experiment uses the MQ-3 sensor to collect concentration data at each sampling point at time t=1s. The sensor readings are converted into concentration values and normalized before being directly compared with the theoretical predictions. The experimental results shown in Figure 18 indicate that different ventilation strategies have a significant impact on droplet transmission. Mixed ventilation achieves a better overall dilution effect by promoting air mixing; displacement ventilation forms a clean air layer in the breathing zone using the thermal stratification principles; while the downward ventilation mode effectively settles droplets through directed airflow, demonstrating the most optimal droplet concentration control effect, which is consistent with the theoretical trend, though there are still differences between the two.

The discrepancies can be attributed to several simplifying assumptions in the theoretical model. In actual ventilation systems, turbulence intensity varies spatially rather than remaining constant, and airflow velocity may decrease along the transport path instead of being uniformly distributed. Moreover, droplets are continuously removed from the airborne phase through gravitational settling and wall deposition, while the emitted droplets exhibit a broad size distribution rather than a single representative size. These practical factors collectively contribute to the discrepancies between theoretical predictions and experimental measurements.

## 5. Discussion and Conclusions

This study systematically explores the dynamics of droplet transmission under the coupled effects of gravity, evaporation, and air resistance through theoretical modeling and numerical analysis. It quantitatively examines the differences in transmission distance and deposition efficiency of droplets generated by typical respiratory activities, such as coughing and speaking. The experimental results indicate that environmental RH and initial droplet size significantly affect the spatial concentration distribution of droplets by altering their evaporation rates and aerodynamic behavior.

Several limitations of the current modeling framework should be acknowledged. First, the droplet transport model is essentially two-dimensional (x–z plane), which does not capture realistic three-dimensional flow structures such as vortex evolution, jet entrainment, turbulent mixing, and puff dynamics that characterize human respiratory jets [35]. Compared with state-of-the-art CFD studies that resolve full 3D Navier–Stokes equations, the present analytical model provides computational efficiency at the expense of spatial resolution. Future work should explore coupling the analytical framework with Reynolds-averaged Navier–Stokes (RANS) or large-eddy simulation (LES) methods to capture three-dimensional effects while retaining the parametric insights provided by the analytical model. Second, human thermal effects—including thermal plumes and buoyancy-induced upward flows surrounding the human body—are well recognized as important mechanisms governing indoor aerosol transport [14], yet are not explicitly modeled here. Under low ventilation rates and low air-change conditions, thermal plumes can significantly alter the vertical transport of exhaled droplets. The omission of thermal effects is justified in the present study because the experimental testbed operates under near-isothermal conditions and the primary focus is on horizontal transport in mechanically ventilated environments. However, for applications involving naturally ventilated or stratified indoor spaces, thermal effects should be incorporated through coupling with temperature field equations. Third, the model adopts a simplified indoor geometry without furniture, occupants, or thermal sources beyond the infected individual; this idealization, while necessary for analytical tractability, means that local concentration predictions do not account for the flow perturbations, recirculation zones, and thermal stratification induced by room furnishings and occupant presence [28]. The ventilation strategies are modeled as uniform velocity fields superposed on the droplet motion (Section 3.3).

The development of the molecular communication testbed represents a methodological contribution that distinguishes this work from previous simulation-based studies. While numerical simulations and epidemiological models have been widely used to investigate droplet transmission, experimental validation in controlled environments remains limited. The testbed developed in this study enables systematic verification of the distance–concentration relationship under various breathing modes and humidity conditions, with $R^{2}$ values exceeding 0.99 for both coughing and sneezing scenarios. Quantitative error metrics—including the $R^{2}$, SSE, and RMSE—are reported in Table 4 and Table 5, providing transparent evaluation of the model’s predictive accuracy. For the coughing scenario, $R^{2}=0.9924$ with RMSE $=6.9\times 10^{-3}$; for the sneezing scenario, $R^{2}=0.9981$ with RMSE $=2.8\times 10^{-3}$. These metrics confirm that the model captures the dominant physical mechanisms governing droplet concentration decay with distance. This experimental approach aligns with the cyber–physical systems paradigm increasingly adopted in engineering informatics research, where the integration of computational models with physical sensing capabilities enhances the reliability of engineering solutions. From a practical perspective, the quantitative relationships established between ventilation parameters, humidity conditions, and droplet dispersion patterns provide actionable guidelines for HVAC system design and operation. The observed increase in the concentration decay rate at higher humidity suggests that humidity may influence horizontal transport under the tested conditions; however, further investigation is required before controlled humidification can be recommended as an engineering control measure. It should be noted that the theoretical model involves certain simplifications (e.g., assuming constant turbulence intensity and uniform airflow distribution), which contribute to deviations between predictions and measurements. Future work will focus on incorporating spatially varying turbulence models and extending the testbed for real-time adaptive ventilation control in smart building applications.

**Figure 19:**
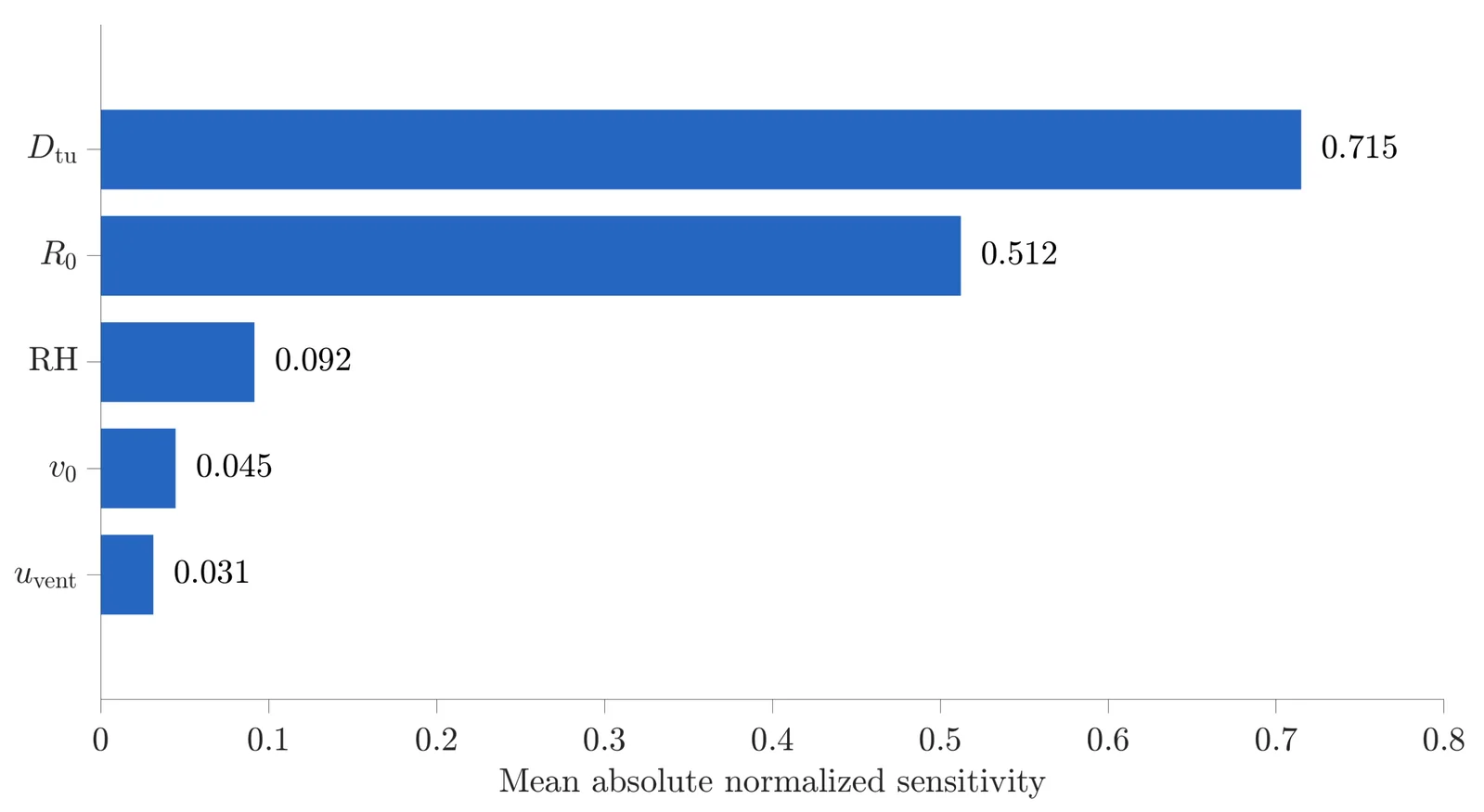
Parameter sensitivity ranking based on the average absolute normalized sensitivity index |S| across all six respiratory modes.

To assess the relative influence of key model parameters, a local normalized sensitivity analysis was performed with ±20% perturbations around baseline values across all six respiratory modes. As shown in Figure 19, the turbulent diffusion coefficient $D_{\mathrm{tu}}$ is the most influential parameter (average normalized sensitivity |S|=0.72), consistent with its dominant role in governing the spatial dispersion of the concentration field. The initial droplet radius $R_{0}$ ranks second (|S|=0.51) through its coupled effects on gravitational settling and evaporation timescale. Relative humidity shows moderate influence (|S|=0.09), while initial velocity and ventilation velocity have comparatively smaller effects within the parameter ranges considered. Ambient temperature is not included because the evaporation rate θ is treated as a constant ($4.2\times 10^{-10}$ m^2^/s) in the present framework.

Additionally, this study incorporates MV and DWV strategies into the theoretical model, enabling the quantitative prediction and evaluation of droplet concentration fields under different ventilation modes. The results confirm that ventilation can alter the distribution of pathogens and droplets, with the appropriate ventilation mode effectively addressing various scenarios. MV and DV can reduce localized droplet accumulation under the modeled ventilation conditions. In particular, DWV alters both short- and long-range droplet transport by directing the emitted droplets downward and facilitating their removal from the breathing zone.

Comparison of different ventilation speeds reveals that increased airflow decreases short-distance exposure to droplets. Furthermore, integrating HVAC systems with filtration and disinfection measures (e.g., HEPA filtration, UVGI) can further enhance protection by effectively removing pathogens during the air intake or exhaust phases, providing multi-level safeguards for indoor air quality.

Several additional limitations warrant mention. The concentration profiles for breathing and speaking (Figure 8) assume strictly horizontal propagation because the modal droplet radii in these activities fall below the 2.5 µm gravitational threshold; in practice, thermal plumes from body heat can deflect the exhaled plume upward—an effect that becomes significant at low ventilation rates and is not captured by the present model. The ventilation strategies are modeled as uniform velocity fields superposed on the droplet motion (Section 3.3). Furthermore, the model does not resolve detailed airflow patterns at the CFD level; ventilation is incorporated through additive velocity components (Section 3.3) and turbulent dispersion is parameterized through an effective diffusion coefficient $D_{\mathrm{tu}}$. This approach captures the net advective–diffusive transport but cannot reproduce localized flow features such as recirculation zones or jet impingement; a detailed comparison of the analytical framework with CFD-resolved airflow simulations is provided in the following paragraph. The study does not consider the resuspension of settled droplets from surfaces, which can serve as a secondary aerosol source [25]. Finally, while the model focuses on physical transport mechanisms, the relationship between exposure dose and infection probability involves biological factors (pathogen viability, host susceptibility) that are beyond the scope of this work.

It is instructive to position the present analytical framework relative to CFD-based simulations of indoor airflow. With sufficiently fine meshes, CFD methods—including RANS, LES, and DNS—resolve the three-dimensional, time-dependent airflow field of a room and reproduce detailed turbulent structures, such as recirculation zones, thermal plumes, and the vortex entrainment of exhaled jets [60,61,62]. In such simulations, droplets are transported as Lagrangian tracers that follow the resolved, non-uniform, time-varying velocity field, so that entrainment by turbulent eddies is an explicit outcome of the computation. By contrast, the present model deliberately replaces the resolved airflow field with two summary quantities: a uniform mean ventilation velocity superposed on the droplet motion (Section 3.3) and an effective turbulent diffusion coefficient $D_{\mathrm{tu}}$ that parameterizes the ensemble-averaged spreading caused by unresolved turbulent fluctuations. These are fundamentally different physical descriptions: $D_{\mathrm{tu}}$ quantifies the statistical dispersion of the droplet cloud, whereas turbulent entrainment is a Lagrangian process in which individual droplets follow instantaneous, spatially non-uniform velocity fluctuations [56]. Consequently, the analytical model is appropriate for time- or ensemble-averaged concentration fields under well-mixed or weakly non-uniform ventilation conditions, but it cannot reproduce instantaneous or localized features such as short-circuiting of the supply jet, recirculation pockets, or transient puff dynamics. As a quantitative guideline, the model is applicable when the turbulent diffusion length scale $\sigma \left(t\right)=\sqrt{4D_{\mathrm{tu}}t}$ is large compared with the characteristic length scale of the unresolved flow non-uniformities, such as the width of the supply jet or the size of the recirculation zones; equivalently, the evaluation points of interest should be far enough from the source that this diffusion length scale exceeds the size of the dominant unresolved flow structures, so that the local airflow details have been smoothed out by turbulent mixing.

Two quantitative consequences follow. First, in the near field of the source, where the exhaled jet dominates and turbulent entrainment governs mixing, a constant-$D_{\mathrm{tu}}$ gradient-diffusion model cannot reproduce the jet-induced concentration fluctuations; comparisons reported in the literature indicate that simplified well-mixed or plume estimates can differ from LES-resolved simulations by an order of magnitude or more in such regimes [62], so CFD is recommended there. Second, in the far field of well-mixed rooms, where the ensemble-averaged concentration is governed by advection and dispersion, gradient-diffusion models provide reasonable approximations—this is the regime in which the present model is intended to operate. The coefficient $D_{\mathrm{tu}}$ should therefore be regarded as an environment-specific effective parameter that absorbs the unresolved eddy effects; for practical room-scale applications, it can be calibrated from CFD simulations or from in situ tracer-gas decay measurements [60,63], in the same spirit as the testbed-based calibration performed in Section 4.2. In a tracer-gas decay test, the well-mixed concentration decays as C(t)=C(0)exp(−λt), where the decay constant λ yields the effective air-exchange rate, and $D_{\mathrm{tu}}$ can subsequently be inferred by matching the analytical concentration profile to the measured spatial decay; alternatively, $D_{\mathrm{tu}}$ can be extracted by fitting the analytical solution to the ensemble-averaged concentration field of a CFD simulation of the specific room. Under the controlled testbed conditions, such calibration yields $R^{2}>0.99$; the corresponding accuracy at room scale cannot be guaranteed a priori and should be verified against CFD predictions or field measurements for each target environment. With such calibration, the analytical model offers a computationally inexpensive complement to CFD: it preserves the explicit parametric dependencies of transmission risk on droplet size, humidity, and ventilation strategy that would be costly to map out by repeated CFD runs. We therefore view the analytical model and CFD as complementary rather than competing tools, and we caution that direct room-scale prediction with the present model requires the airflow-specific calibration described above.

The findings of this study introduce an innovative theoretical tool for assessing the risk of indoor viral transmission. When combined with room-specific airflow information obtained from CFD simulations or in situ tracer-gas measurements, the quantitative model can provide engineering guidance for optimizing ventilation system design and implementing precise epidemic prevention measures. Future research will focus on developing a multi-scale experimental platform to validate and calibrate the model, ultimately creating a decision-support system for controlling airborne diseases in real-world scenarios.

## Figures and Tables

**Table 1:** Solute volume fraction ($\Phi_{0}$) in droplets under different scenarios.

Scenario	Solute Volume Fraction ($\Phi_{0}$)	Basis
Normal breathing/speaking droplets	1–2%	Saliva-based guidance [43]
Coughing or droplets with more airway mucus	2–3%	Upper respiratory mucus mixture [40]
Deep lung droplets (alveolar)	0.5–1%	Alveolar fluid analysis [24]

**Table 2:** Distribution of droplet size and initial concentration under different respiratory activities.

Respiratory Activity	Initial Velocity (m/s)	Initial Particle Number	Main Size Range (μm)
Breathing	1.3	100–340	0.1–5 (Mode: 0.8) [46]
Speaking	5	500–1000	0.5–50 (Mode: 1.6) [46]
Coughing	10	1100–2000	0.5–500 (Mode: 1.5, 74) [48]
Sneezing	30	3000–5000	0.5–2000 (Mode: 74.4, 360.1) [48]

**Table 3:** Parameter settings.

Parameters	Description	Value
$\Phi_{0}$	Initial volume fraction of aerosol	0.02
$\rho_{d}$	Density of aerosol	997 kg/m^3^
$\rho_{a}$	Air density	1.225 kg/m^3^
*K*	Air resistance coefficient	0.47
η	Air viscosity coefficient	$1.85\times 10^{-5}$ kg/ms
γ	Water activity coefficient	1
$D_{\mathrm{tr}}$	Laminar diffusion coefficient	$10^{-4}$ m^2^/s
$D_{\mathrm{tu}}$	Turbulent diffusion coefficient	0.24 m^2^/s
$R_{\mathrm{Rx}}$	Radius of person’s receiving area	0.1 m

**Table 4:** Fitting parameters and performance evaluation metrics for two breathing modes.

Method	*v*	*N*	$A_{1}$	$A_{2}$	$A_{3}$	$A_{4}$	$A_{5}$	$R^{2}$	SSE	RMSE
Cough	10	$10^{15}$	$4.0\times 10^{-5}$	$2.26\times 10^{-3}$	$4.2\times 10^{-4}$	5.90	1.66	0.9924	$1.904\times 10^{-3}$	$6.9\times 10^{-3}$
Sneeze	30	$10^{16}$	$3.8\times 10^{-5}$	$1.89\times 10^{-3}$	$5.5\times 10^{-4}$	8.72	2.15	0.9981	$3.136\times 10^{-4}$	$2.8\times 10^{-3}$

**Table 5:** Fitting parameters and performance evaluation metrics under three humidity conditions.

RH	$A_{1}$	$A_{2}$	$A_{3}$	$A_{4}$	$A_{5}$	$R^{2}$	SSE	RMSE
40%	$4.0\times 10^{-5}$	$1.36\times 10^{3}$	$7.01\times 10^{-4}$	5.90	1.66	0.9924	$1.904\times 10^{-3}$	$6.9\times 10^{-3}$
60%	$3.5\times 10^{-5}$	$2.29\times 10^{3}$	$4.67\times 10^{-4}$	6.35	1.42	0.9957	$8.76\times 10^{-4}$	$4.2\times 10^{-3}$
80%	$3.1\times 10^{-5}$	$4.54\times 10^{3}$	$2.5\times 10^{-4}$	6.81	1.28	0.9973	$4.32\times 10^{-4}$	$3.1\times 10^{-3}$

**Table 6:** Parameters under different ventilation conditions.

Ventilation Mode	Fan Configuration	Airflow Direction	Ventilation Velocity
No Ventilation	None	None	0 m/s
Mixed Ventilation	Two sides at the top of the box (1 intake, 1 exhaust)	Horizontal flow	0.5 m/s
Displacement Ventilation	Two sides at the bottom of the box (intake), top (exhaust)	Vertical rising	0.3 m/s
Downward Ventilation	Top of the box (intake), two sides at the bottom (exhaust)	Vertical descending	0.3 m/s

## Data Availability

Data are not available for privacy.

## Abbreviations

The following abbreviations and symbols are used in this manuscript:

R(t)	Time-dependent droplet radius (m)
$R_{0}$	Initial droplet radius (m)
$R_{\mathrm{ev}}$	Equilibrium (droplet nucleus) radius (m)
RH	Relative humidity
θ	Evaporation constant (m^2^/s)
$\Phi_{0}$	Initial solute volume fraction
γ	Water activity coefficient
$\rho_{d}$	Droplet density (kg/m^3^)
$\rho_{a}$	Air density (kg/m^3^)
η	Dynamic viscosity of air (kg/(m·s))
*K*	Drag coefficient
Rep	Particle Reynolds number
Ref	Flow Reynolds number
$v_{0}$	Initial droplet velocity (m/s)
$v_{x}\left(t\right),v_{z}\left(t\right)$	Velocity components in x and z directions (m/s)
$s_{x}\left(t\right),s_{z}\left(t\right)$	Displacement in x and z directions (m)
*g*	Gravitational acceleration (m/s^2^)
*h*	Emission height (m)
$t_{\mathrm{sed}}$	Sedimentation time (s)
$t_{\mathrm{ev}}$	Evaporation time (s)
$D_{\mathrm{tr}}$	Laminar diffusion coefficient (m^2^/s)
$D_{\mathrm{tu}}$	Turbulent diffusion coefficient (m^2^/s)
*N*	Number of aerosol particles per emission
C(x,y,z,t)	Droplet concentration (particles/m^3^)
$Q_{\mathrm{Rx}}$	Cumulative viral exposure (particles)
MV	Mixed ventilation
DV	Displacement ventilation
DWV	Downward ventilation

## Appendix A

This paper presents the simulation results of airborne concentration and the virus exposure levels of susceptible person under different respiratory activities and ventilation strategies. These results are based on the theoretical model we proposed, which is detailed in Section 2. The following is the derivation process of $v_{x}\left(t\right)$ and $v_{z}\left(t\right)$ in Section 2.

When Rep>0.1, $t_{\mathrm{sed}}\leq t_{\mathrm{ev}}$, and R(t) is used to represent the particle radius, the mass of the droplet can be expressed as $m=\frac{4}{3}\pi \rho_{d}R{\left(t\right)}^{3}$. In the x direction, the force situation for droplets is given by

(A1) $$F_{x}\left(t\right)=\frac{4}{3}\pi \rho_{d}R{\left(t\right)}^{3}\frac{dv_{x}\left(t\right)}{dt}=-\frac{1}{2}\rho_{a}K\pi v_{x}^{2}\left(t\right)R{\left(t\right)}^{2}.$$

Due to the evaporation effect, which indirectly affects the quality of the droplets by changing their radius, we will substitute $R\left(t\right)=\sqrt{R_{0}^{2}-t\theta \left(1-\mathrm{RH}\right)}$ into the equation and rearrange it to obtain

(A2) $$\frac{dv_{x}\left(t\right)}{v_{x}^{2}\left(t\right)}=-\frac{3\rho_{a}Kdt}{8\rho_{d}\sqrt{R_{0}^{2}-t\theta \left(1-\mathrm{RH}\right)}}.$$

Integrating the left side, we obtain

(A3) $$\frac{1}{v_{x}\left(t\right)}=\frac{3\rho_{a}K}{8\rho_{d}}\int \frac{dt}{\sqrt{R_{0}^{2}-t\theta \left(1-\mathrm{RH}\right)}}+C_{1}.$$

Use the substitution method for the integral on the right side, we have $u=R_{0}^{2}-t\theta \left(1-\mathrm{RH}\right),\;dt=-\frac{du}{\theta (1-\mathrm{RH})}$. Therefore,

(A4) $$\int \frac{dt}{\sqrt{R_{0}^{2}-t\theta \left(1-\mathrm{RH}\right)}}=-\frac{1}{\theta (1-\mathrm{RH})}\int \frac{du}{\sqrt{u}}=-\frac{2\sqrt{R_{0}^{2}-t\theta \left(1-\mathrm{RH}\right)}}{\theta (1-\mathrm{RH})}+C_{2}.$$

Substituting back into the original equation gives

(A5) $$-\frac{1}{v_{x}\left(t\right)}=\frac{3\rho_{a}K\sqrt{R_{0}^{2}-t\theta \left(1-\mathrm{RH}\right)}}{4\rho_{d}\theta \left(1-\mathrm{RH}\right)}+C_{3}.$$

Assuming the initial condition $v_{x}\left(0\right)=v_{0}$, we find

(A6) $$C_{3}=-\frac{1}{v_{0}}-\frac{3\rho_{a}KR_{0}}{4\rho_{d}\theta \left(1-\mathrm{RH}\right)}.$$

Thus, the final solution becomes

(A7) $$v_{x}\left(t\right)=\frac{1}{\frac{3\rho_{a}K}{4\rho_{d}\theta \left(1-\mathrm{RH}\right)}\left(R_{0}-\sqrt{R_{0}^{2}-t\theta \left(1-\mathrm{RH}\right)}\right)+\frac{1}{v_{0}}}.$$

Next, for the z direction, considering the weight of the droplet, the force situation for droplets in the z direction can be expressed as

(A8) $$F_{z}\left(t\right)=\frac{4}{3}\pi R{\left(t\right)}^{3}\rho_{d}\frac{dv_{z}\left(t\right)}{dt}=\frac{4}{3}\pi R{\left(t\right)}^{3}g\left(\rho_{a}-\rho_{d}\right)-\frac{1}{2}\rho_{a}K\pi v_{z}^{2}\left(t\right)R{\left(t\right)}^{2}.$$

Substituting in *m* and R(t), we obtain

(A9) $$\frac{dv_{z}\left(t\right)}{dt}=\frac{g(\rho_{a}-\rho_{d})}{\rho_{d}}-\frac{3\rho_{a}Kv_{z}^{2}\left(t\right)}{8\rho_{d}R\left(t\right)}.$$

Let $\alpha =\frac{g(\rho_{a}-\rho_{d})}{\rho_{d}},\;\beta =\frac{3\rho_{a}K}{8\rho_{d}}$. Due to the complexity of the equation, we perform a Taylor expansion of R(t) around t=0, retaining the first-order terms to obtain

(A10) $$R\left(t\right)\approx R_{0}+\frac{dR}{dt}{|}_{t=0}t=R_{0}-\frac{\theta (1-\mathrm{RH})}{2R_{0}}t.$$

Substituting $R\left(t\right)\approx R_{0}-\epsilon t$ (where $\epsilon =\frac{\theta (1-\mathrm{RH})}{2R_{0}}\to 0$), we integrate

(A11) $$\int \frac{dv_{z}\left(t\right)}{\alpha -\beta \frac{v_{z}^{2}\left(t\right)}{R_{0}}}=\int dt.$$

Integrating results in

(A12) $$\frac{1}{\sqrt{\frac{\alpha \beta }{R_{0}}}}\tanh^{-1}\left(\sqrt{\frac{\beta }{\alpha R_{0}}}v_{z}\left(t\right)\right)=t+C_{4}.$$

At t=0, $v_{z}\left(0\right)=0$ leads to $C_{4}=0$. Thus, the z direction velocity $v_{z}\left(t\right)$ is given by

(A13) $$v_{z}\left(t\right)=\sqrt{\frac{8g\left(\rho_{a}-\rho_{d}\right)R_{0}}{3\rho_{a}K}}\tanh\left(\sqrt{\frac{3g\left(\rho_{a}-\rho_{d}\right)\rho_{a}K}{8\rho_{d}^{2}R_{0}}}t\right).$$

For $t_{\mathrm{sed}}>t_{\mathrm{ev}}$, where $0<t\leq t_{\mathrm{ev}}$, the expressions for R(t), $v_{x}\left(t\right)$, and $v_{z}\left(t\right)$ are similar to those in Equations (7) and (13). For the interval $t_{\mathrm{ev}}<t\leq t_{\mathrm{sed}}$, denoted as $R_{\mathrm{ev}}$, the force situation in the x direction is given by

(A14) $$F_{x}\left(t\right)=m\frac{dv_{x}\left(t\right)}{dt}=-\frac{1}{2}\rho_{a}Kv_{x}^{2}\left(t\right)\pi R_{\mathrm{ev}}^{2}.$$

Proceeding with variable separation, we obtain

(A15) $$\frac{dv_{x}\left(t\right)}{v_{x}^{2}\left(t\right)}=-\frac{3\rho_{a}K}{8\rho_{d}R_{\mathrm{ev}}}dt.$$

Integrating yields

(A16) $$\frac{1}{v_{x}\left(t\right)}=\frac{3\rho_{a}Kt}{8\rho_{d}R_{\mathrm{ev}}}+C_{5}.$$

Thus, we obtain

(A17) $$v_{x}\left(t\right)=\frac{8\rho_{d}R_{\mathrm{ev}}}{3\rho_{a}Kt+8\rho_{d}R_{\mathrm{ev}}C_{5}}.$$

By substituting the initial condition Rep>0.1 at $t=t_{\mathrm{ev}}$, for $0<t\leq t_{\mathrm{ev}}$ with $v_{x}\left(t_{\mathrm{ev}}\right)=v_{x}\left(t_{\mathrm{ev}}\right)$ for $t_{\mathrm{ev}}<t\leq t_{\mathrm{sed}}$, $C_{5}$ can be determined.

The force in the z direction is given by

(A18) $$F_{z}\left(t\right)=m\frac{dv_{z}\left(t\right)}{dt}=\frac{4}{3}\pi R_{\mathrm{ev}}^{3}g\left(\rho_{a}-\rho_{d}\right)-\frac{1}{2}\rho_{a}Kv_{z}^{2}\left(t\right)\pi R_{\mathrm{ev}}^{2}.$$

Proceeding with variable separation, we obtain

(A19) $$\frac{dv_{z}\left(t\right)}{dt}=\frac{g(\rho_{a}-\rho_{d})}{\rho_{d}}-\frac{3\rho_{a}K}{8\rho_{d}R_{\mathrm{ev}}}v_{z}^{2}\left(t\right).$$

Let $A=\frac{g(\rho_{a}-\rho_{d})}{\rho_{d}}$, $B=\frac{3\rho_{a}K}{8\rho_{a}R_{\mathrm{ev}}}$. The equation can be rewritten as

(A20) $$\frac{dv_{z}\left(t\right)}{A-Bv_{z}^{2}\left(t\right)}=dt.$$

For $\rho_{a}<\rho_{d}$, let A=−|A|; then, the equation becomes

(A21) $$\frac{dv_{z}\left(t\right)}{-\left|A\right|-Bv_{z}^{2}\left(t\right)}=dt.$$

Utilizing the integral formula gives

(A22) $$-\sqrt{|A|B}\;\arctan\left(\sqrt{\frac{B}{\left|A\right|}}v_{z}\left(t\right)\right)=t+C_{6}.$$

The solution is given by

(A23) $$v_{z}\left(t\right)=\sqrt{\frac{\left|A\right|}{B}}\tan\left(\sqrt{|A|B}\left(C_{6}-t\right)\right),$$

where $A=\frac{g(\rho_{a}-\rho_{d})}{\rho_{d}}$ and $B=\frac{3\rho_{a}K}{8\rho_{d}R_{\mathrm{ev}}}$. By substituting the initial condition Rep>0.1 at $t=t_{\mathrm{ev}}$ for $0<t\leq t_{\mathrm{ev}}$ with $v_{z}\left(t_{\mathrm{ev}}\right)=v_{z}\left(t_{\mathrm{ev}}\right)$ for $t_{\mathrm{ev}}<t\leq t_{\mathrm{sed}}$, $C_{6}$ can be determined.

When Rep≤0.1, $t_{\mathrm{sed}}\leq t_{\mathrm{ev}}$, where R(t) represents the flight radius, the force in the x direction is given by

(A24) $$F_{x}\left(t\right)=\frac{4}{3}\pi R{\left(t\right)}^{3}\rho_{d}\frac{dv_{x}\left(t\right)}{dt}=-6\pi \eta R\left(t\right)v_{x}\left(t\right).$$

Proceeding with variable separation, we obtain

(A25) $$\ln v_{x}\left(t\right)=\frac{9\eta \ln(R_{0}^{2}-t\theta \left(1-\mathrm{RH}\right))}{2\rho_{d}\theta \left(1-\mathrm{RH}\right)}+C_{7},$$

where $C_{7}$ is a constant of integration. At t=0, substituting $v_{x}\left(0\right)=v_{0}$ yields

(A26) $$\ln v_{0}=\frac{9\eta \ln(R_{0})}{\rho_{a}\theta \left(1-\mathrm{RH}\right)}+C_{7}.$$

Thus, we find the velocity equation for $v_{x}\left(t\right)$ as

(A27) $$\ln v_{x}\left(t\right)=\frac{9\eta \ln(R_{0}^{2}-t\theta \left(1-\mathrm{RH}\right))}{2\rho_{d}\theta \left(1-\mathrm{RH}\right)}+\ln v_{0}-\frac{9\eta \ln(R_{0})}{\rho_{d}\theta \left(1-\mathrm{RH}\right)}.$$

Taking the exponential of both sides yields

(A28) $$v_{x}\left(t\right)=v_{0}{\left(\frac{R_{0}^{2}-t\theta \left(1-\mathrm{RH}\right)}{R_{0}^{2}}\right)}^{\frac{9\eta }{2\rho_{d}\theta \left(1-\mathrm{RH}\right)}}.$$

The force acting on the particles in the z direction is given by

(A29) $$F_{z}\left(t\right)=\frac{4}{3}\pi R{\left(t\right)}^{3}\rho_{d}\frac{dv_{z}\left(t\right)}{dt}=\frac{4}{3}\pi R{\left(t\right)}^{3}g\left(\rho_{a}-\rho_{d}\right)+6\pi \eta R\left(t\right)v_{z}\left(t\right).$$

Rearranging yields

(A30) $$\frac{dv_{z}\left(t\right)}{dt}-\frac{9\eta v_{z}\left(t\right)}{2\rho_{d}\left(R_{0}^{2}-t\theta \left(1-\mathrm{RH}\right)\right)}=\frac{g(\rho_{a}-\rho_{d})}{\rho_{d}}.$$

This is a first-order non-linear differential equation, which can be described using an integrating factor as

(A31) $$\begin{array}{cc} \phi (t) & =\exp\left(-\int \frac{9\eta }{2\rho_{d}\left(R_{0}^{2}-t\theta \left(1-\mathrm{RH}\right)\right)}dt\right)\\ \; & =\exp\left(\frac{9\eta }{2\rho_{d}\theta \left(1-\mathrm{RH}\right)}\ln\left|R_{0}^{2}-t\theta \left(1-\mathrm{RH}\right)\right|\right)\\ \; & ={\left(R_{0}^{2}-t\theta \left(1-\mathrm{RH}\right)\right)}^{\frac{9\eta }{2\rho_{d}\theta \left(1-\mathrm{RH}\right)}}. \end{array}$$

To find the solution, we have

(A32) $$\begin{array}{cc} v_{z}\left(t\right) & =\frac{1}{\phi (t)}\left(\int \frac{g(\rho_{a}-\rho_{d})}{\rho_{d}}\phi \left(t\right)dt+C_{8}\right)\\ \; & ={\left(R_{0}^{2}-t\theta \left(1-\mathrm{RH}\right)\right)}^{\frac{-9\eta }{2\rho_{d}\theta \left(1-\mathrm{RH}\right)}}\\ \; & \times \left(\frac{g(\rho_{a}-\rho_{d})}{\rho_{d}}\int {\left(R_{0}^{2}-t\theta \left(1-\mathrm{RH}\right)\right)}^{\frac{9\eta }{2\rho_{d}\theta \left(1-\mathrm{RH}\right)}}dt+C_{8}\right). \end{array}$$

The integral computes to yield

(A33) $$\int {\left(R_{0}^{2}-t\theta \left(1-\mathrm{RH}\right)\right)}^{\frac{9\eta }{2\rho_{d}\theta \left(1-\mathrm{RH}\right)}}dt=-\frac{{\left(R_{0}^{2}-t\theta \left(1-\mathrm{RH}\right)\right)}^{\frac{9\eta }{2\rho_{d}\theta \left(1-\mathrm{RH}\right)}+1}}{\left(\frac{9\eta }{2\rho_{d}\theta \left(1-\mathrm{RH}\right)}+1\right)\theta \left(1-\mathrm{RH}\right)}+C_{9}.$$

Thus, we consolidate as

(A34) $$\begin{array}{cc} v_{z}\left(t\right) & =\frac{-g(\rho_{a}-\rho_{d})}{\rho_{d}\theta \left(1-\mathrm{RH}\right)\left(\frac{9\eta }{2\rho_{d}\theta \left(1-\mathrm{RH}\right)}+1\right)}\\ \; & \times \left(R_{0}^{2}-t\theta \left(1-\mathrm{RH}\right)\right)+C_{10}{\left(R_{0}^{2}-t\theta \left(1-\mathrm{RH}\right)\right)}^{\frac{-9\eta }{2\rho_{d}\theta \left(1-\mathrm{RH}\right)}}. \end{array}$$

By substituting $v_{z}\left(0\right)=0$, we find $C_{10}=\frac{2g\left(\rho_{a}-\rho_{d}\right)R_{0}^{2}}{2\rho_{a}\theta \left(1-\mathrm{RH}\right)+9\eta }R_{0}^{\frac{9\eta }{\rho_{d}\theta \left(1-\mathrm{RH}\right)}}$; thus,

(A35) $$\begin{array}{cc} v_{z}\left(t\right) & =\frac{2g(\rho_{a}-\rho_{d})}{2\rho_{a}\theta \left(1-\mathrm{RH}\right)+9\eta }\\ \; & \times \left(R_{0}^{2}{\left(\frac{R_{0}^{2}}{R_{0}^{2}-t\theta \left(1-\mathrm{RH}\right)}\right)}^{\frac{9\eta }{2\rho_{d}\theta \left(1-\mathrm{RH}\right)}}-\left(R_{0}^{2}-t\theta \left(1-\mathrm{RH}\right)\right)\right). \end{array}$$

For $t_{\mathrm{sed}}>t_{\mathrm{ev}}$, where $0<t\leq t_{\mathrm{ev}}$, similarly, the forms of $v_{x}\left(t\right)$ and $v_{z}\left(t\right)$ correspond to those in Equations (28) and (35). In the range $t_{\mathrm{ev}}<t<t_{\mathrm{sed}}$, the force situation in the x direction is given by

(A36) $$F_{x}\left(t\right)=\frac{4}{3}\pi R_{\mathrm{ev}}^{3}\rho_{d}\frac{dv_{x}\left(t\right)}{dt}=-6\pi \eta R_{\mathrm{ev}}v_{x}\left(t\right).$$

Proceeding with variable separation, we obtain

(A37) $$\frac{dv_{x}\left(t\right)}{v_{x}\left(t\right)}=-\frac{9\eta }{2R_{\mathrm{ev}}^{2}\rho_{d}}dt.$$

Integrating gives

(A38) $$\ln|v_{x}\left(t\right)|=-\frac{9\eta }{2R_{\mathrm{ev}}^{2}\rho_{d}}t+\ln\left|C_{11}\right|.$$

From the equations previously obtained, we have

(A39) $$v_{x}\left(t\right)=C_{11}\exp\left(-\frac{9\eta t}{2R_{\mathrm{ev}}^{2}\rho_{d}}\right).$$

By substituting the initial condition Rep≤0.1 at $t=t_{\mathrm{ev}}$ for $0<t\leq t_{\mathrm{ev}}$ with $v_{x}\left(t_{\mathrm{ev}}\right)=v_{x}\left(t_{\mathrm{ev}}\right)$ for $t_{\mathrm{ev}}<t\leq t_{\mathrm{sed}}$, $C_{11}$ can be determined.

The force situation in the z direction is given by

(A40) $$F_{z}\left(t\right)=\frac{4}{3}\pi R_{\mathrm{ev}}^{3}\rho_{d}\frac{dv_{z}\left(t\right)}{dt}=\frac{4}{3}\pi R_{\mathrm{ev}}^{3}g\left(\rho_{a}-\rho_{d}\right)+6\pi \eta R_{\mathrm{ev}}v_{z}\left(t\right).$$

Rearranging yields the first-order non-linear differential equation:

(A41) $$\frac{dv_{z}\left(t\right)}{dt}-\frac{9\eta }{2\rho_{d}R_{\mathrm{ev}}^{2}}v_{z}\left(t\right)=\frac{g(\rho_{a}-\rho_{d})}{\rho_{d}}.$$

The solution is

(A42) $$v_{z}\left(t\right)=C_{12}\exp\left(\frac{9\eta t}{2\rho_{d}R_{\mathrm{ev}}^{2}}\right)-\frac{2gR_{\mathrm{ev}}^{2}\left(\rho_{a}-\rho_{d}\right)}{9\eta }.$$

By substituting the initial condition Rep≤0.1 at $t=t_{\mathrm{ev}}$ for $0<t\leq t_{\mathrm{ev}}$ with $v_{z}\left(t_{\mathrm{ev}}\right)=v_{z}\left(t_{\mathrm{ev}}\right)$ for $t_{\mathrm{ev}}<t\leq t_{\mathrm{sed}}$, $C_{12}$ can be determined.
